# Elevated Na/H exchanger 1 (*SLC9A1*) emerges as a marker for tumorigenesis and prognosis in gliomas

**DOI:** 10.1186/s13046-018-0923-z

**Published:** 2018-10-17

**Authors:** Xiudong Guan, Lanxin Luo, Gulnaz Begum, Gary Kohanbash, Qingkun Song, Aparna Rao, Nduka Amankulor, Baoshan Sun, Dandan Sun, Wang Jia

**Affiliations:** 1 Department of Neurosurgery, Beijing Tiantan Hospital, Capital Medical University, No. 6 Tiantan Xili, Dongcheng District, Beijing, 100050 China; 2 Chinese National Clinical Research Center for Neurological Diseases, Beijing, China; 3 Beijing Neurosurgical Institute, Beijing, China; 4 Chinese Glioma Genome Atlas Network, Beijing, 100050 China; 5 Department of Neurological Surgery, University of Pittsburgh, Pittsburgh, PA USA; 6 Department of Science and Technology, Beijing Shijitan Hospital, Capital Medical University, Beijing, China; 7 School of Traditional Chinese Materia Medica, Shenyang Pharmaceutical University, Shenyang, China; 8 Pólo Dois Portos, Instituto National de Investigação Agrária e Veterinária, I.P., Quinta da Almoinha, Dois Portos, Portugal; 9 Department of Neurology, University of Pittsburgh, 7016 Biomedical Science Tower 3 3501 Fifth Ave., Pittsburgh, PA 15260 USA; 10 Pittsburgh Institute for Neurodegenerative Disorders, University of Pittsburgh, Pittsburgh, PA USA

**Keywords:** Angiogenesis, Anti-PD1 therapy, Immunosuppression, NHE1 inhibitor HOE642, Tumor-associated microglia/macrophage

## Abstract

**Background:**

Sodium/hydrogen exchanger 1 (NHE1), encoded by the *SLC9A1* gene (SoLute Carrier family 9A1) in humans, is the main H^+^ efflux mechanism in maintaining alkaline intracellular pH (pH_i_) and Warburg effects in glioma. However, to date, there are no clinical studies exploring pharmacological inhibition of NHE1 protein in cancer treatment. In this study, we investigated NHE1 expression in gliomas and its relationship with glioma clinical outcome.

**Methods:**

The Chinese Glioma Genome Atlas (CGGA) dataset containing transcriptome sequencing data of 325 glioma samples and the Cancer Genome Atlas (TCGA) with 698 glioma mRNAseq data were analyzed in this study. Mouse SB28 and GL26 intracranial syngeneic glioma models in C57BL/6 J mice were established to investigate NHE1 expression and impact of NHE1 protein inhibition with its inhibitor HOE642 on tumorigenesis and anti-PD1 therapy. Tumor angiogenesis, immunogenicity, and progression were assessed by immunofluorescence staining and flow cytometric profiling.

**Results:**

Analysis of *SLC9A1* mRNA expression in two data sets, CGGA and TCGA, reveals significantly higher *SLC9A1* mRNA levels in higher grade gliomas. The *SLC9A1* mRNA expression was especially enriched in isocitrate dehydrogenase (IDH)1/2 wild-type glioblastoma (GBM) and in mesenchymal glioma subtypes. Worsened survival probabilities were correlated with the elevated *SLC9A1* mRNA levels in gliomas. The underlying mechanisms include promoting angiogenesis, and extracellular matrix remodeling. Increased *SLC9A1* mRNA expression was also associated with tumor-associated macrophage accumulation. NHE1 inhibitor HOE642 reduced glioma volume, invasion, and prolonged overall survival in mouse glioma models. Blockade of NHE1 protein also stimulated immunogenic tumor microenvironment via activating CD8 T-cell accumulation, increasing expression of interferon-gamma (*Ifng*), and sensitized animals to anti-PD-1 therapy.

**Conclusion:**

Our findings strongly suggest that NHE1 protein emerges as a marker for tumorigenesis and prognosis in glioma. Blocking NHE1 protein is a novel strategy for adjuvant anti-cancer therapies.

**Electronic supplementary material:**

The online version of this article (10.1186/s13046-018-0923-z) contains supplementary material, which is available to authorized users.

## Background

Cancer cells rely on oxidative glycolysis by increasing glucose uptake and lactate production rather than mitochondrial respiration even in the presence of oxygen and fully functioning mitochondria, a process known as Warburg effect [1–3]. An alkaline intracellular pH (pH_i_) is the driving force for glycolytic metabolism [4, 5]. Sodium/hydrogen exchanger 1 (NHE1), encoded by the *SLC9A1* gene (SoLute Carrier family 9A1) in humans [6], is the main H^+^ efflux mechanism in maintaining alkaline pH_i_ in cancer cells [7]. Several new studies illustrate that NHE1 promotes tumor cell proliferation in gastric cancer [8], hepatocellular carcinoma [9], ovarian cancer [10], non-small cell lung cancer [11] and breast cancer invasiveness and progression [12, 13]. These findings suggest that NHE1 protein has emerged as an important therapeutic target against tumorigenesis and progression. However, to date, there are no clinical studies exploring pharmacological inhibition of NHE1 protein in cancer treatment [4, 14].

Others’ and our studies demonstrate that NHE1 protein transports H^+^ efflux in exchange of Na^+^ influx for maintaining pH_i_ of ~7.3–7.5 in human GBM cells in vitro [15, 16]. We also reported that NHE1 protein plays a critical role in proliferation and invasion of cultured human primary glioma cells [17]. Most importantly, our recent study shows that temozolomide (TMZ) treatment induces glioma cells to upregulate NHE1 protein expression in an intracranial mouse syngeneic glioma model bearing SB28-GFP (non-immunogenic) or GL26-Cit tumors (immunogenic) [18]. Combining the TMZ chemotherapy with NHE1 protein inhibitor HOE642 (cariporide) was more effective in reducing glioma tumor growth and improving median survival than the TMZ monotherapy [18]. These findings motivated us to conduct this informatics study to systemically analyze *SLC9A1* mRNA expression in two data sets, the Chinese Glioma Genome Atlas (CGGA) dataset containing transcriptome sequencing data of 325 glioma samples and The Cancer Genome Atlas (TCGA) mRNAseq data of 698 gliomas. Our study reveals that significantly higher *SLC9A1* mRNA levels were detected in all grades of gliomas. Its expression is enriched in isocitrate dehydrogenase (IDH)1/ 2 wild-type glioblastoma and in mesenchymal glioma subtypes. Worsened survival probabilities were correlated with the elevated *SLC9A1* mRNA levels in gliomas. Pharmacological blockade of NHE1 protein reduced TAMs, decreased associated cytokines, enhanced CD8 accumulation, increased the expression of interferon-gamma in the T cells, and sensitized animals to anti-PD-1 therapy. Our findings strongly suggest that NHE1 protein emerges as a marker for tumorigenesis and prognosis in glioma, blocking NHE1 protein is a novel strategy for adjuvant anti-cancer therapies.

## Methods

### Data collection

Clinical and molecular information from the Chinese Glioma Genome Atlas (CGGA) dataset contained transcriptome sequencing data of 325 glioma samples generated by Illumina HiSeq platform and approved by Capital Medical University Institutional Review Board (IRB). The methodology of detecting IDH mutation status was described in the previous study [19]. The Cancer Genome Atlas (TCGA) mRNAseq data of 698 gliomas, ranging from WHO grade II to grade IV, was downloaded from public databases (https://portal.gdc.cancer.gov/). RNA-seq data with specific tumor anatomic structures in GBM, identified by H&E staining, was from Ivy Glioblastoma Atlas Project (http://glioblastoma.alleninstitute.org/).

### Bioinformatics analysis

RNA-seq data of CGGA (FPKM value), TCGA (FPKM value), and IVY GBM dataset (FPKM value) were log transformed before analysis. For the expression analysis, cases were classified into different group according to WHO Grade classification, IDH mutant status, and TCGA molecular subtype [20, 21]. Before the Kaplan Meier survival analysis, cases were divided into two groups (high expression and low expression) based on the average value of *SLC9A1* mRNA expression. The biological functions of the related genes were explored by GO analysis in DAVID Bioinformatics Resources 6.8 [22]. After Spearman correlation analysis, gene ontology (GO) analysis of the most correlated genes was constructed using “pheatmap” package in R. The biological phenotype was verified by gene set enrichment analysis (GSEA) [23]. The correlation between endothelial cell and *SLC9A1* mRNA expression in human glioma tissue was analyzed by microenvironment cell populations (MCP) counter [24]. The association between metagene of immune cells and *SLC9A1* was performed by gene set variation analysis (GSVA), as described in the previous study [19, 25, 26]. The gene lists of endothelial cell and immune cells were downloaded from previously published articles [19, 25, 27].

### Materials

Dulbecco’s Modified Eagle Medium (DMEM/HEPES, Cat #12430-054) and Penicillin/Streptomycin (Cat #15240062) were from Gibco (Carlsbad, CA). Fetal bovine serum (FBS) and G418 sulfate were obtained from Invitrogen (Carlsbad, CA). Rabbit antibody against β-actin (Cat #4970S) was from Cell Signaling (Beverly, MA). Rabbit antibody against NHE1 (Cat #ab67314) and rat antibody against CD8 (Cat #ab22378) were from Abcam Ltd. (Cambridge, MA). Rat anti-mouse CD31 (Cat #550274) was from BD Pharmingen (San Jose, CA). Rabbit anti-ionized calcium-binding adapter molecule 1 (iba1) was from Wako (Richmond, VA). PerCP/Cy5.5-CD45, PE-P2RY12, BV421-TGFβ, BV605-TNFα, APC/780-IL-1β, PE/Cy7-IL10, PerCP/Cy5.5-CD8a, APC/Cy7-CD4, Alexa Fluor 700-CD25, PE-FoxP3, BV605-PD-1, PE-Gr-1, APC-NK1.1, and Pacific Blue Granzyme B were obtained from Biolegend (San Diego, CA). eFluor 450-CD16/32, PE/Cy7-CD206, APC-IFNγ was purchased from eBioscience (San Diego, CA). APC-IL-6 were obtained from thermos fisher (Waltham, MA). BUV737-CD11b, Alexa Flour 700-CD86 were obtained from BD Biosciences (San Jose, CA). PE-Ym1were from Abcam Ltd. (Cambridge, MA). Rabbit anti-Laminin (Cat #L9393) and cariporide (HOE642) was purchased from Sigma Chemicals (St. Louis, MO). Anti-mouse PD-1 (RMP1-14) and IgG2a isotype control were from BioXcell (West Lebanon, NH).

### Human brain tissue samples

Paraformaldehyde fixed tissue from surgically removed CNS tumors were collected at the Department of Pathology, University of Pittsburgh Medical Center, following approval by the institutional review boards. All tumors were classified according to WHO diagnostic criteria.

### Cell cultures and authentication

The mouse glioma SB28-GFP cells were derived as described previously [28]. The SB28-GFP or GL26 cells were seeded in DMEM/HEPES containing 10% heat-inactivated FBS, 2 mM L-glutamine, and 1× Penicillin/streptavidin. Cultures were passaged approximately every 4 days with fresh medium at a density of 10^6^ cells/75cm^2^ in a culture flask (<25 passages used in the study).

### Intracranial syngeneic mouse glioma models

All animal experiments were approved by the University of Pittsburgh Institutional Animal Care and Use Committee and performed in accordance with the National Institutes of Health Guide for the Care and Use of Laboratory Animals.

C57BL/6 mice (female, 7–8 weeks old) were anesthetized with 2% isoflurane. Animals under anesthesia were placed into a stereotactic frame and a single midline incision was made to expose the cranium. A hole was drilled into the cranium above the left cerebral hemisphere using a precision power drill equipped with a fine bit at the following coordinates from bregma: + 0.5 mm AP, + 2.1 mm ML, and − 3.2 mm DV. Using aseptic technique, upon exposing the underlying dura, 5–10 × 10^4^ SB28-GFP or GL26 glioma cells in 2 μL of serum-free DMEM was injected into the right striatum using a micro-pump injector and a 5-μl Hamilton syringe equipped with a 33-gauge needle for 4 min at a rate of 500 nl/min. Cells were allowed to settle for 5 min followed by slow needle withdrawal. Ketofen (2 mg/kg, i.p.) was administrated once prior to surgery and daily for 2 days after the surgery and then daily if animals exhibit signs of pain. Animals were then allowed to recover in their cages under a heat lamp and access to water and wet chow.

### Drug treatment regimen in syngeneic mouse glioma models

Starting 5 days after tumor cell implantation (d.p.i.), mice were randomly assigned to each treatment group and received the therapy for 5 consecutive days: vehicle control (Veh, 1.25% DMSO in PBS, 10 ml/kg/day, i.p.) or NHE1 inhibitor HOE642 (H, 0.25 mg/kg, twice a day, i.p.) treatment at 5–9 d.p.i.. For the immunotherapy, mice were treated either with isotype IgG2a (10 mg/kg/day, i.p.), anti-PD-1 antibody (10 mg/kg/day, i.p.) at 8, 10, and 12 d.p.i, or H at 5–9 d.p.i. followed with anti-PD-1 at 8, 10, and 12 d.p.i.

### Animal survival test

Tumor bearing animals were monitored daily for signs of pain, discomfort or neurological impairment. Signs of chronic pain, such as hunched posture, weight loss, absence of grooming behavior, and of neurological impairment, like seizures, weakness, difficulty walking, an inability to right themselves, circling behavior, and unusual aggressiveness or timidity were used to infer tumor development. In tumor cell injected mice, a loss of 20% body weight, severe neurological impairment, or major loss in body scoring index (<2.0 on a 5-point scale) were used as the humane endpoint. All other surviving mice were sacrificed at 90 days after glioma cell injection.

### Evaluation of glioma tumor in syngeneic mouse glioma models

At the termination point, animals were anesthetized with 3% isoflurane in 70% N_2_O and 30% O_2_ and exhibited no toe and tail reflexes. Animals were transcardially perfused with 0.9% saline solution using a 40 ml syringe followed by a solution of 4% paraformaldehyde (PFA) in PBS (pH 7.4). Brains were harvested and stored in 4% PFA at 4 °C overnight, then stored in 30% sucrose for protection. Coronal tissue sections (25 μm thick) were made using a vibratome (Leica SM 2010R, Buffalo Grove, IL). To measure xenograft tumor size, the GFP-positive tumor area in each brain section (an interval of 150 μm) were selected and measured using ImageJ software. Tumor volume was calculated as described previously (multiplying the sum of tumor area measurement by the height including section thickness and the z gap between slices) [29]. To quantify tumor invasion, fluorescent images were captured with a 4× objective using Nikon TE 300 inverted epifluorescence microscope at 488 nm laser. The GFP-positive SB28 tumor mass was identified and the tumor-invaded area recognized where: i) isolated GFP-positive glioma cells at the tumor border are mixed with surrounding GFP-negative normal brain cells, or ii) gliomas display pseudopodia-like structures. Two parameters were used to evaluate tumor invasion: maximal invasive distance which was measured by the distance between the farthest GFP-positive glioma cells (arrows in Fig. 4c) from the bulk tumor mass edge (dashed line in Fig. 4c); percentage of invasive area was determined by [invasive area / bulk tumor mass area plus invasive area] × 100, as described previously [30]. Five to seven brain sections per mouse were used for the quantification and the means of parameters were obtained for statistical analysis.

### Immunofluorescence staining

Fixed brain sections (4% PFA) were mounted on microscope slides. Sections were then incubated with a blocking solution (0.3% Triton X-100 and 3% goat serum in PBS) for 60 min at room temperature (RT) and probed with primary antibodies (rabbit antibody against NHE1, 1:200; rabbit antibody against Iba1, 1:200; rat antibody against CD31, 1:50; rat antibody against CD8, 1:100) overnight at 4 °C. After rinsing in PBS three times for 15 min, tissue sections were incubated with respective secondary antibodies conjugated to Alexa Fluor® 546 (1:200 dilution) for 1 h at RT. Sections were then rinsed and incubated either with To-pro-3 iodide (1: 1000) or DAPI (1:1000) for 15 min at RT and mounted with Vectashield mounting medium. Negative controls of brain sections were stained with respective secondary antibodies alone (Additional file 1: Figure S1). Fluorescence images were captured with a Leica DMIRE2 inverted confocal laser-scanning microscope under the 40× oil immersion objective lens, with the excitation at 488, 546 and 630 nm and the emission fluorescence was recorded at 490–525 nm, 556–573 nm, and 650–750 nm, respectively. Details of analysis were described in the Additional file 1: Supplementary methods.

### Flow cytometry

Once mice were anesthetized with isoflurane, transcardial perfusion was performed with 0.9% NaCl and brain tissues were dissociated with the Neural Tissue Dissociation Kit per manufacturer’s instruction (Miltenyi Biotech, Gladbach, Germany). The procedure of isolation for flow cytometry was performed as previously described [31]. Cells were suspended in Hanks balanced salt solution (HBSS) containing FBS. Cells were stained with anti-mouse BUV737-CD11b, PerCP/Cy5.5-CD45, PE-P2RY12, eFluor 450-CD16/32, and PE/Cy7-CD206 to assess TAM infiltration. For cytokines, cells were stained with BUV737-CD11b, PerCP/Cy5.5-CD45, PE-P2RY12, BV421-TGFβ, BV605-TNFα, APC-IL-6, APC/780-IL-1β, and PE/Cy7-IL10. For T-Cell profiling, cells were stained with PerCP/Cy5.5-CD8a, Apc/Cy7-CD4, PE-FoxP3, Alexa Fluor 700-CD25, BV605-PD-1, and APC-IFNγ. For detecting infiltration of peripheral blood mononuclear (PBMCs), cells were stained with BUV737-CD11b, PerCP/Cy5.5-CD45, PE-Gr-1, APC-NK1.1, and Pacific Blue Granzyme B as described [31]. Samples were acquired with a BD LSRII instrument (at least 100,000 events were recorded for each sample) and analyzed with Flow Jo (Tree Star) software.

### Statistical analysis

The prognostic value of NHE1 was estimated by Univariate and multivariate Cox proportional hazard model analysis. Patients with missing information were excluded from corresponding analysis. The results were expressed as the mean ± standard error of the mean (SEM) or standard deviation (SD). Statistical significance was determined by Student’s t-test for single comparison or one-way analysis of variance (ANOVA) for multiple comparisons. Correlations between continuous variables was evaluated by Spearman correlation analysis. Overall survival of patients or mice was evaluated using Kaplan-Meier analysis and compared with a two-sided log-rank test. All statistical analysis was performed using IBM SPSS Statistical software (version 24.0, Armonk, NY: IBM Corp), R project (version 3.4.1, https://www.r-project.org/), and Prism 7 (GraphPad Software, Inc). A *P*-value < 0.05 was considered statistically significant. *N* values represent the number of independent in vivo or in vitro experiments.

## Results

### High expression of *SLC9A1* mRNA was associated with malignancy in gliomas

**Fig. 1:**
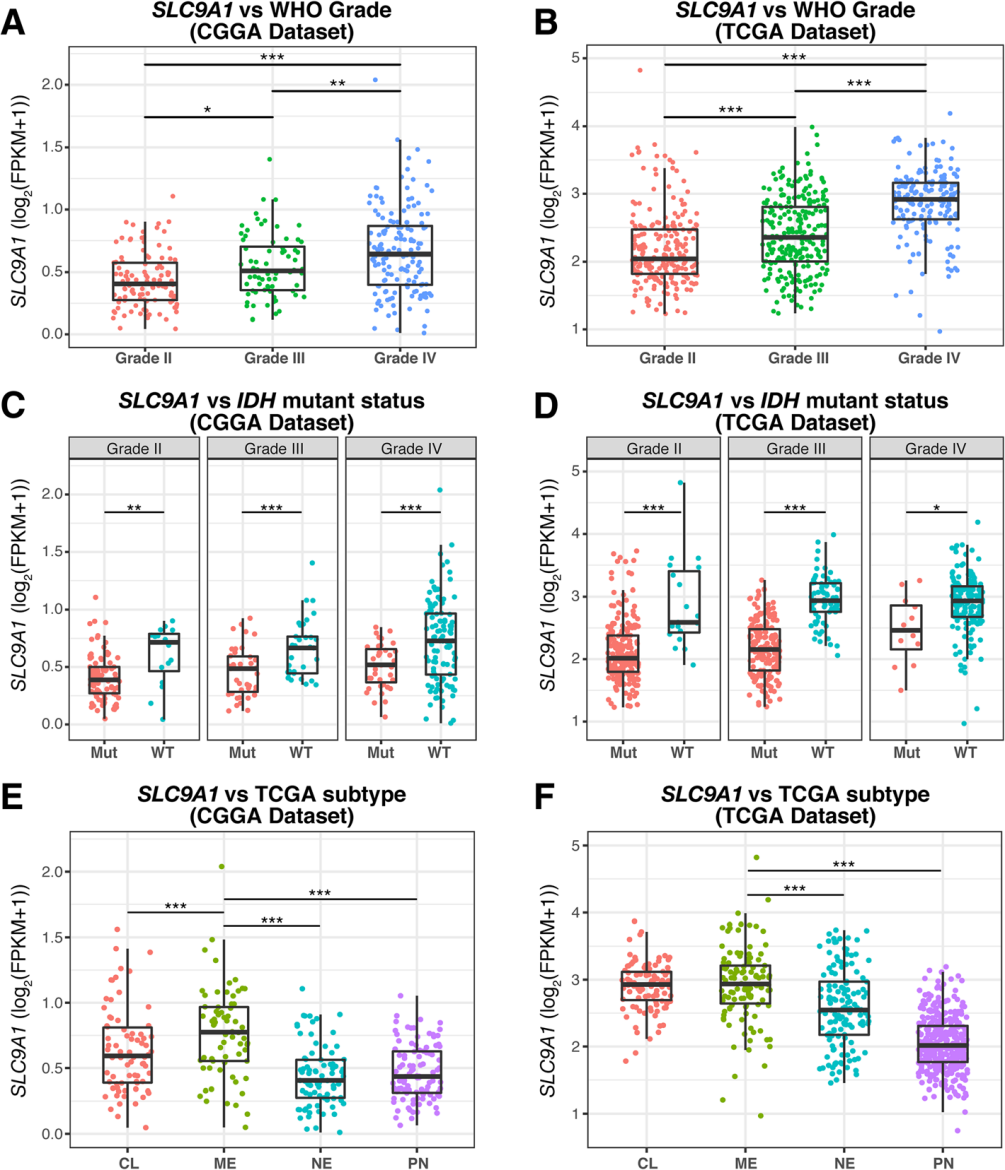
Overexpression of *SLC9A1* mRNA is associated with malignancy in gliomas. **a**, **b** *SLC9A1* mRNA expression in different WHO Grade gliomas from CGGA and TCGA datasets, respectively. Data are mean ± SD. *, *p* < 0.05; **, *p* < 0.01; ***, *p* < 0.001. **c**, **d** *SLC9A1* mRNA expression in different grade gliomas according to IDH status. Mut, IDH mutant status; WT, IDH wildtype status. Data are mean ± SD. *, *p* < 0.05; **, *p* < 0.01; ***, *p* < 0.001. **e**, **f** *SLC9A1* mRNA expression according to TCGA subtype classification. CL, Classical; ME, Mesenchymal; NE, Neural; PN, Proneural. Data are mean ± SD. ***, *p* < 0.001

Analysis of the RNAseq data revealed that *SLC9A1* mRNA expression was higher in glioblastoma than WHO grade II and III gliomas in CGGA cohort (Fig. 1a) as well as in TCGA cohort (Fig. 1b). Considering of IDH mutation status as one of important biomarkers for tumorigenesis and prognosis [32, 33], we analyzed association of *SLC9A1* mRNA expression with the genotypes of *IDH* mutations as a sub-classifier. Figure 1c and d show that both CGGA and TCGA datasets exhibited significantly higher *SLC9A1* mRNA expression in the IDH-wildtype (WT) than the IDH-mutant (Mut) gliomas in all grades. Among the different molecular subtypes defined by TCGA network [21], the high level of *SLC9A1* mRNA expression was significantly enriched in mesenchymal (ME) subtype in both CGGA and TCGA datasets (Fig. 1e, f). *SLC9A1* mRNA expression levels of mesenchymal subclass is significantly different from the classical (CL), neural (NE), and proneural (PN) subtypes in TCGA cohort (Fig. 1e). These results imply that elevated *SLC9A1* mRNA expression is associated with malignancy in gliomas.

### High *SLC9A1* mRNA expression in gliomas is associated with worse survival prognosis

**Fig. 2:**
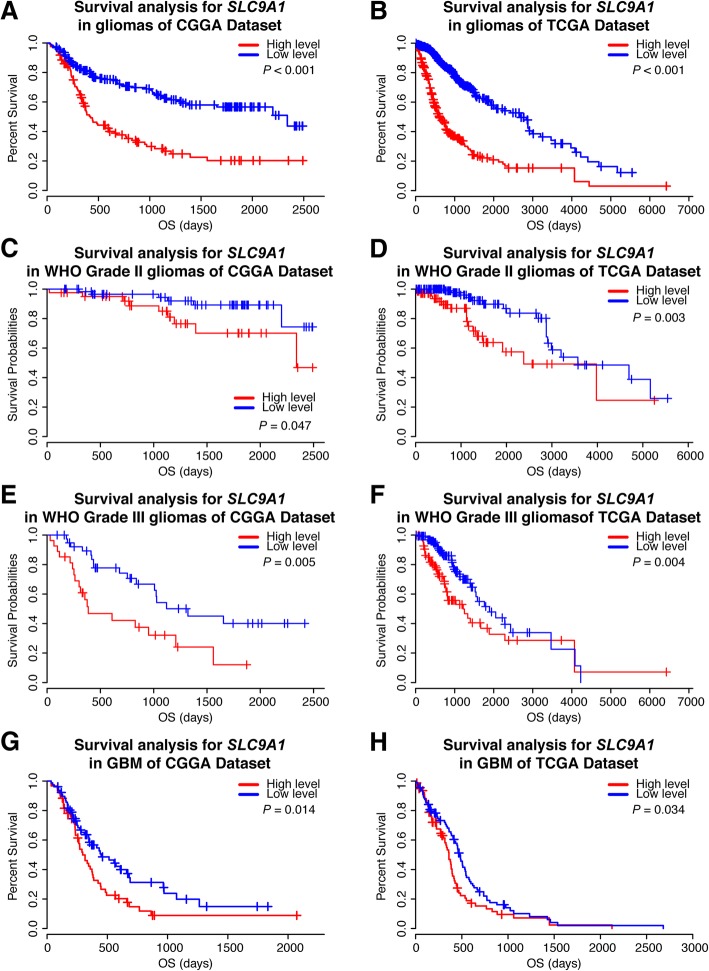
High expression of *SLC9A1* mRNA correlates with poor outcomes in glioma patients. **a**, **b** Kaplan-Meier survival analysis of the whole WHO grades of gliomas according to *SLC9A1* mRNA expression. **c**, **d** Kaplan-Meier survival analysis of WHO Grade II gliomas. **e**, **f** Kaplan-Meier survival analysis of the WHO Grade III gliomas. **g**, **h** Kaplan-Meier survival analysis of the GBM

We then performed the Kaplan-Meier analysis and Cox proportional hazard model to assess the prognostic values of *SLC9A1* mRNA expression in gliomas. Figure 2a and b show that patients with high *SLC9A1* mRNA expression exhibited significantly shorter overall survival in the whole gliomas of the CGGA cohort as well as in the TCGA cohort. Regarding the heterogeneity of different grade gliomas, the similar findings were detected in Grade II and III gliomas (Fig. 2c–f). Importantly, the Kaplan-Meier survival analysis revealed that GBM patients with higher *SLC9A1* mRNA expression was associated with a poor outcome (Fig. 2g and h). Furthermore, our univariate and multivariate cox analysis indicate *SLC9A1* mRNA elevation as an independent factor for outcome of glioma patients (Table 1). Taken together, these data strongly suggest that *SLC9A1* (NHE1 protein) may serve as a new prognostic biomarker for gliomas.

**Table 1:** Univariate and Multivariate Analysis of clinical prognostic parameters in CGGA cohort

Variable	Total No.	Univariate analysis^a^		Multivariate analysis^a, b^	
		*P* value	HR (95% CI)	*P* value	HR (95% CI)
*NHE1* expression	310	<0.001	3.400 (2.427–4.761)	0.033	1.578 (1.038–2.399)
Age at diagnosis	310	<0.001	1.038 (1.023–1.054)	0.995	–
Gender	310	0.345	0.847 (0.600–1.195)	0.461	–
WHO Grade	310	<0.001	3.477 (2.716–4.452)	<0.001	2.888 (2.204–3.784)
*IDH* mutation	310	<0.001	0.244 (0.170–0.350)	0.017	0.599 (0.393–0.914)

^a^Cox regression analysis

^b^Further adjusting age and gender

Neither the Cancer Proteome Atlas nor The Human Protein Atlas contains NHE1 protein score or expression data in glioma tissues. To determine NHE1 protein expression in human gliomas, we performed immunofluorescence staining for NHE1 protein on paraformaldehyde-fixed sections of five human glioma tissues (from Grade II to Grade IV, Additional file 2: Table S1). Additional file 1: Figure S2 show that grade II–III astrocytoma exhibited low levels of NHE1 protein expression (**arrows**). In contrast, abundant NHE1 immunoreactive fluorescence staining signals were detected in cytoplasma and membrane compartments of grade IV glioma tumor cells (**arrows**). NHE1 immunoreactive fluorescence staining intensity increased with increasing tumor malignancy. Collectively, these data are consistent with our analysis of SLC9A1 expression of TCGA and CGGA dataset, suggesting that high levels of NHE1 protein expression are associated with malignant progression of gliomas.

### Elevated *SLC9A1*mRNA expression in gliomas was associated with angiogenesis in biological process analysis

**Fig. 3:**
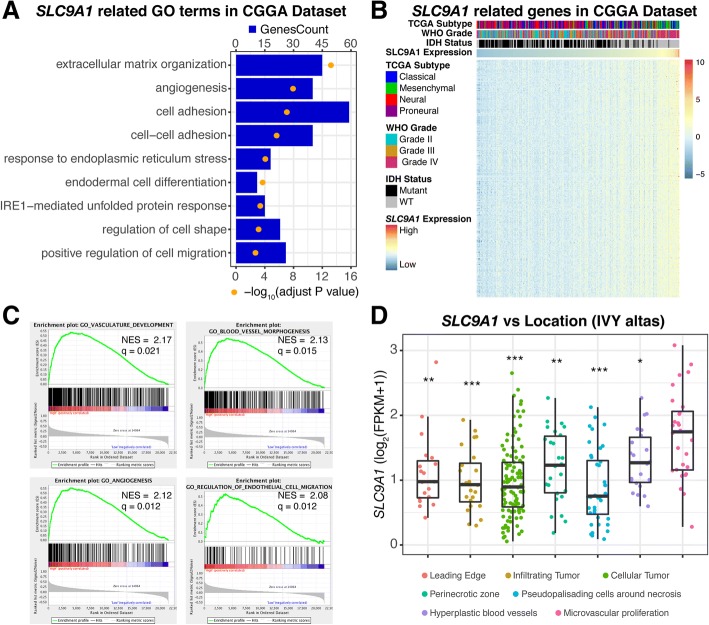
*SLC9A1* mRNA expression is related with angiogenesis in gene ontology analysis. **a** Gene ontology (GO) analysis of positively related biological process shows that NHE1-associated genes are enriched in extracellular matrix organization, angiogenesis and cell adhesion in CGGA datasets. **b** The heatmaps display *SLC9A1*-related genes in CGGA cohorts. **c** Gene Set Enrichment Analysis (GSEA) indicated a stronger angiogenesis phenotype in the case with high NHE1 expression in CGGA dataset. q, adjust *p* value. **d** NHE1 mRNA expression was highest in the tumor area of microvascular proliferation compared with other areas in the gliomas from the Ivy Glioblastoma Atlas Project. *, *P* < 0.05; **, *P* < 0.01; ***, *p* < 0.001

To further understand the biological roles of NHE1 expression in gliomas, we analyzed correlation between *SLC9A1* mRNA and 1020 genes in the CGGA cohort or 1332 genes in the TCGA cohort by Pearson correlation analysis (with Pearson coefficient |R| > 0.4). The biological functions of the related genes were detected by gene ontology analysis in DAVID Bioinformatics Resources 6.8 [22]. Figure 3a illustrates that the genes positively correlated with *SLC9A1* mRNA expression in gliomas were enriched in processes for extracellular matrix organization, angiogenesis, and cell adhesion, with adjusted *p*-value in an increasing order in the CGGA dataset. A similar trend was detected in the TCGA cohort (Additional file 1: Figure S3A). In addition, the heatmap showed that there are top 300 genes which are positively correlated with *SLC9A1* mRNA expression in each dataset (Fig. 3b and Additional file 1: Figure S3B). Gene set enrichment analysis (GSEA) further indicated that glioma with various *SLC9A1* mRNA expression levels presented distinct status of angiogenesis. When dividing cases of CGGA dataset into high *SLC9A1* mRNA expression and low expression groups (above or below the median expression), stronger angiogenic phenotypes were exhibited in the high *SLC9A1* mRNA expression cases (Fig. 3c). We further analyzed the data from the Ivy Glioblastoma Atlas Project in respect of *SLC9A1* mRNA expression in the different regions of tumors. As shown in Fig. 3d, the highest *SLC9A1* mRNA expression was detected in the tumor region with robust microvascular proliferation, which is consistent with the results of GO terms analysis (Fig. 3a and c).

### Inhibition of NHE1 protein with its potent inhibitor HOE642 reduced glioma tumor volume and invasion in mouse syngeneic intracranial glioma model

**Fig. 4:**
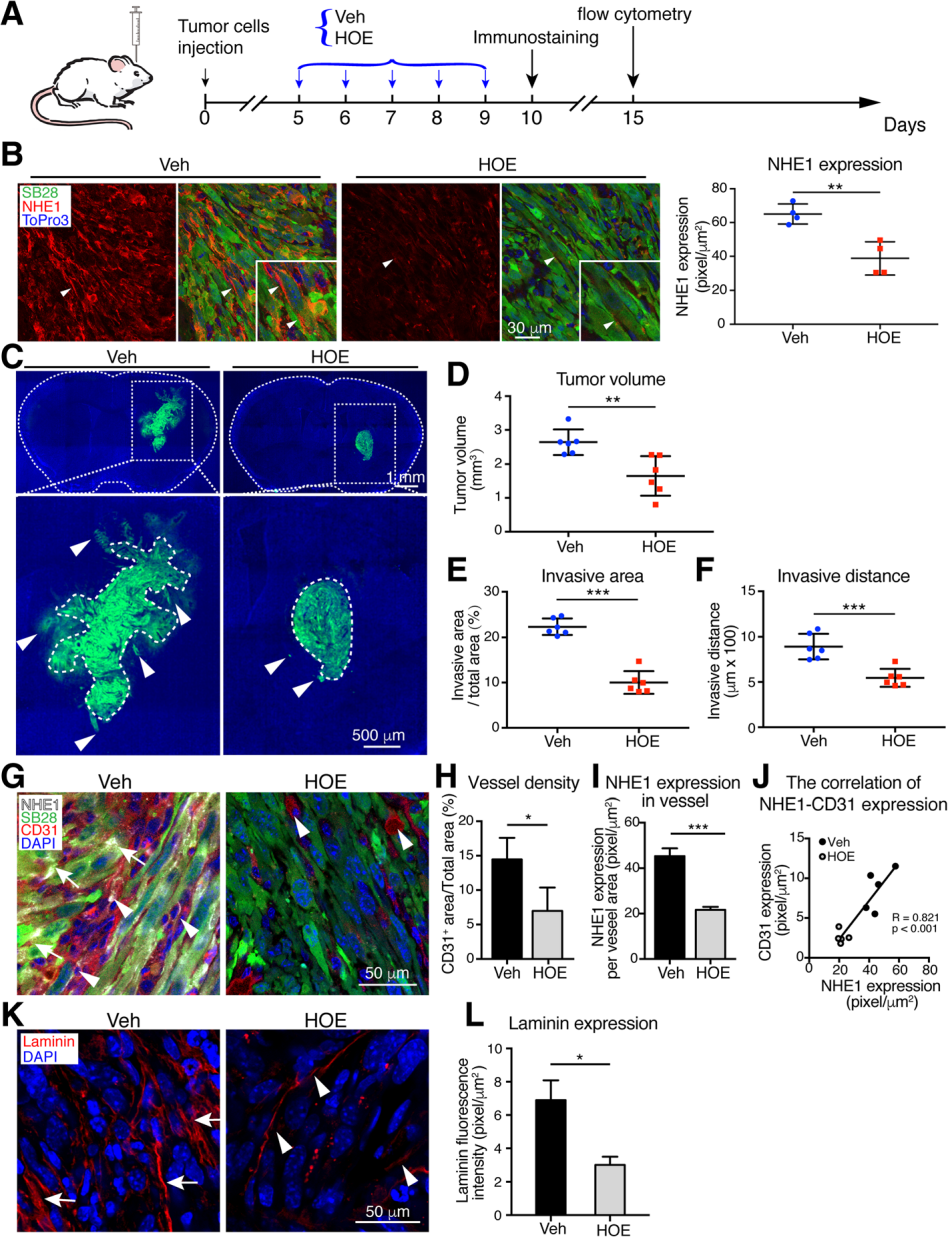
Pharmacological inhibition of NHE1 protein decreases tumor volume, invasion, and angiogenesis. **a** Experimental protocol. SB28-GFP cells (40,000) or GL26 cells (100,000) were injected in the right striatum of C57BL/6 J mice. Starting 5 days after implantation (d.p.i.), mice received either vehicle (Veh, 1.25% DMSO in PBS, 10 ml/kg/day) or HOE (0.5 mg/kg/day, ip) for 5 consecutive days. Mice were sacrificed at 10 d.p.i. for immunostaining and at 15 d.p.i. for flow cytometry. **b** Representative immunostaining of fixed brain sections (25 μm) for NHE1 protein expression in SB28-GFP tumor. Data are means ± SEM from four independent experiments. **, *p* < 0.01. **c** Shown SB28-GFP tumors of Vehicle group and HOE group at 10 d.p.i., respectively. Arrows, the farthest isolated GFP-positive glioma cells; Dashed line, bulk tumor area border. **d** HOE treatment decreased SB28-GFP tumor volumes at 10 d.p.i. Data are means ± SD from six independent experiments. **, *p* < 0.01. **e**, **f** Tumor invasion was quantified by measuring the maximal invasion distance, as well as the invasive front as a percentage of total tumor. Data are means ± SEM from six independent experiments. ***, *p* < 0.005. **g** Representative confocal images of SB28-GFP tumor sections (25 μm) immunostained for NHE1 and endothelial cell marker CD31. Arrows: NHE1 expression in tumor cells, arrowhead: NHE1 expression in the CD31^+^ vessels. **h** Data summary by vessel area/total area. Data are means ± SEM from four independent experiments. *, *p* < 0.05. **i** NHE1 expression in vessel area. Data are means ± SEM from four independent experiments. ***, *p* < 0.001. **j** Regression correlation analysis of NHE1 expression in vessel area and CD31 expression in tumor. **k** Representative confocal images of ECM protein laminin staining in SB28-GFP tumor. Arrows: increased accumulation of laminin; Arrowheads: less laminin accumulation. **l** Summary data of laminin fluorescence intensity. Data are mean ± SEM, from seven independent experiments. *, *p* < 0.05

We then investigated whether inhibition of NHE1 protein with its potent inhibitor, HOE642, would reduce tumor progression in C57BL/6 J mice bearing syngeneic intracranial glioma. As shown in Fig. 4a, C57BL/6 J mice were transplanted with SB28-GFP or GL26 cells, and subsequently received vehicle (Veh) control (DMSO) or HOE642 treatment (i.p) for five consecutive days (5–9 d.p.i.). Lower NHE1 protein levels were observed in SB28-GFP tumors in the HOE642-treated mice than in the Veh-treated control mice (Fig. 4b). HOE642 treatment decreased the tumor volume by ~28% compared with the Veh-control group (Fig. 4c and d). Inhibition of NHE1 protein also reduced the invasive ability of SB28-GFP (Fig. 4e and f).

The biological process analysis of GO terms suggests a role of NHE1 protein in tumor vasculature remodeling. Analysis of the endothelial cell score by microenvironment cell populations (MCP)-counter in CGGA dataset showed that the endothelial MCP score was positively correlated with *SLC9A1* mRNA expression level (Additional file 1: Figure S4A). This led us to probe for endothelial marker protein CD31 expression in the SB28-GFP tumor areas in mice. Tumor tissues exhibited high cerebral vessel density (Fig. 4g; Additional file 1: Figure S4B). In contrast, HOE642-treated mice exhibited reduced CD31^+^ vessel density (Fig. 4h; Additional file 1: Figure S4C). To further understand roles of NHE1 protein in tumor vessel remodeling, we conducted co-staining of CD31 protein and NHE1 protein. NHE1 protein was detected in CD31^+^ vessels (**arrowhead**) and SB28-GFP^+^ tumor cells (**arrows**) in the Veh-treated tumors (Fig. 4g). Interestingly, HOE642-treated SB28 tumors show nearly absence of CD31^+^ vessels or NHE1 protein expression (Fig. 4i). CD31 expression in tumor was positively correlated with NHE1 protein expression in the vessels (Fig. 4j). Laminin is an important ECM component and the major constituent of basement membrane which is involved in tumor extracellular matrix remodeling [34]. Glioma cells secrete laminin for tumor cell adhesion and migration [35]. We assessed the expression of laminin in the Veh-control and HOE642-treated tumors by immunofluorescence staining analysis. As shown in Fig. 4k, abundant expression of laminin protein appears as a cross-linked mesh network in the Veh-treated glioma tumor (**arrows**). Interestingly, in the HOE642-treated tumors, laminin expression was significantly reduced and the network at the vessel structure was not abundant (**arrowheads**, Fig. 4k;). Quantification of laminin fluorescence intensity signals revealed that the Veh-treated brain tumors exhibited ~56% higher laminin expression than the HOE642-treated brain tumors (*p* < 0.05, Fig. 4l). These results clearly indicate that inhibition of NHE1 protein via HOE642 may decrease tumor invasion via reducing laminin secretion and ECM remodeling. Taken together, these findings suggest that endothelial NHE1 protein plays a role in endothelial proliferation and tumor vasculature remodeling.

### Pharmacological blockade of NHE1 protein reduces tumor-associated macrophage activation

**Fig. 5:**
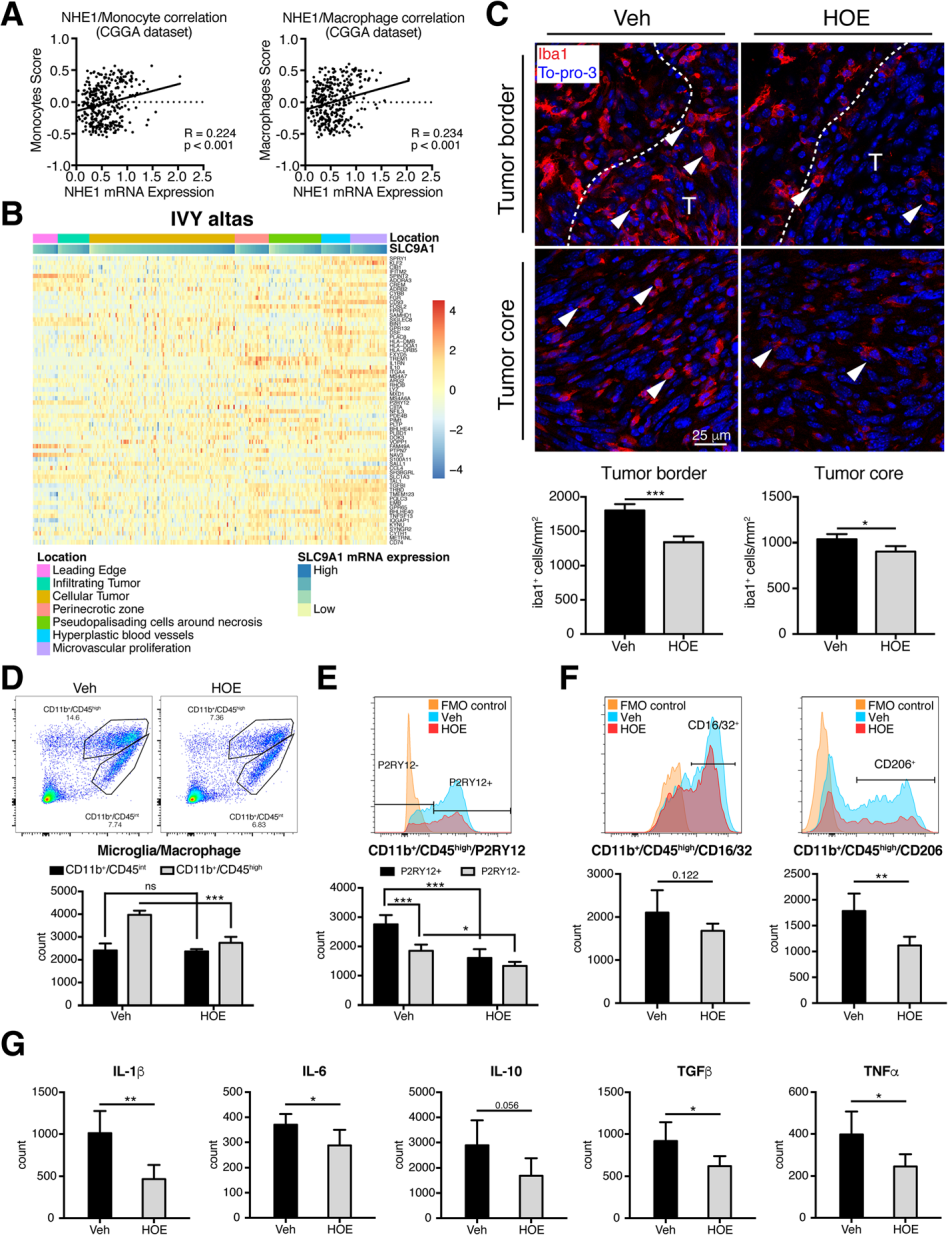
Inhibition of NHE1 deceases angiogenesis by reducing TAMs and associated cytokines. **a** Regression correlation analysis of NHE1 mRNA expression and monocytes/macrophages metagene score in CGGA dataset. **b** TAMs related genes were enriched in the areas of microvascular proliferation and hyperplastic blood vessels from the Ivy Glioblastoma Atlas Project. **c** Infiltration of Iba1^+^ cell in the tumor borders and tumor cores of SB28-GFP glioma-bearing mice. T, tumor. Data are means ± SEM from four independent experiments. * *p* < 0.05. ***, *p* < 0.001. **d** Representative flow cytometric profile showing gating strategy of microglia (CD11b^+^/CD45^int^) and macrophages (CD11b^+^/CD45^high^) in the same protocol as described in Fig. 4a. Data are means ± SD from five independent experiments. ns, no significance; ***, *p* < 0.001. **e** Representative flow-cytometry plot of TAMs stained for P2RY12 in fluorescence minus one (FMO) control, Veh, and HOE. Data are means ± SD from five independent experiments. *, *p* < 0.05; ***, *p* < 0.001. **f** Inflammatory profile of TAMs stained for CD16/32 and CD206. Data are means ± SD from five independent experiments. **, *p* < 0.01. **g** Flow-cytometry plots are shown in Additional file 1: Figure S6. Bar graphs show data summary of cytokines for TAMs. Data are means ± SD from five independent experiments. *, *p* < 0.05. **, *p* < 0.01

Metagene scores of monocytes and macrophages (CGGA cohort) showed a positive correlation with increased *SLC9A1* mRNA expression in the human glioma tissues (Fig. 5a). Analysis of IVY glioblastoma dataset shows that 66 marker genes of tumor associated macrophages/microglia (TAMs) were mainly enriched in the tumor region of microvascular proliferation (Fig. 5b) [27]. We recently also reported that NHE1 protein plays an important role in protumoral stimulation of TAMs [17]. Here, we investigated impact of blocking NHE1 protein on infiltration of TAMs in mouse glioma models. The infiltrations of Iba1^+^ cells were detected in both tumor border and core regions in the Veh-control (Fig. 5c) and HOE642 treatment significantly reduced Iba1^+^ cell infiltration ((Fig. 5c). Using flow-cytometric profiling, we detected that HOE642 treatment reduced the count of macrophages (CD11b^+^/CD45^high^ cells), but not microglia population (CD11b^+^/CD45^int^ cells) (Fig. 5d) [36]. P2RY12, a well-known specific microglia marker, was used to further differentiate microglia TAMs vs peripheral bone marrow TAMs [27]. Figure 5e shows the amount of resident microglia TAMs (CD11b^+^/CD45^high^/P2RY12^+^ cells) was higher than the peripheral infiltrated TAMs (CD11b^+^/CD45^high^/P2RY12^−^ cells). The HOE642-treated tumors exhibited reduced microglia TAMs and peripheral infiltrated TAMs, with more pronounced drop in microglia TAMs (Fig. 5e).

We next investigated the inflammatory profiling of TAMs. No significant changes of pro-inflammatory CD16/32^+^ cells were detected in microglia TAMs (Additional file 1: Figure S5A) or total TAMs in the HOE642-treated tumors (Fig. 5f), except a reduced CD16/32^+^ population was found in the peripheral infiltrated TAMs (Additional file 1: Figure S5B). However, significant reduction of pro-tumoral CD206^+^ cells was observed in all classified TAMs (Fig. 5f; Additional file 1: Figure S5A and B). Furthermore, we found microglia TAMs and total TAMs from the HOE642-treated tumors exhibit reduced cytokine expression (such as IL-1β, IL-6, TGFβ, and TNFα in Fig. 5g; Additional file 1: Figures S5A, B, and S6). But few cytokines were decreased in peripheral TAMs from the HOE642-treated tumors (Additional file 1: Figure S5A and B). Taken together, our data show that HOE642 treatment reduced infiltration and activation of microglia and peripheral TAMs.

### Pharmacological blockade of NHE1 enhances the anti-tumor immunity and anti-PD-1 therapy

**Fig. 6:**
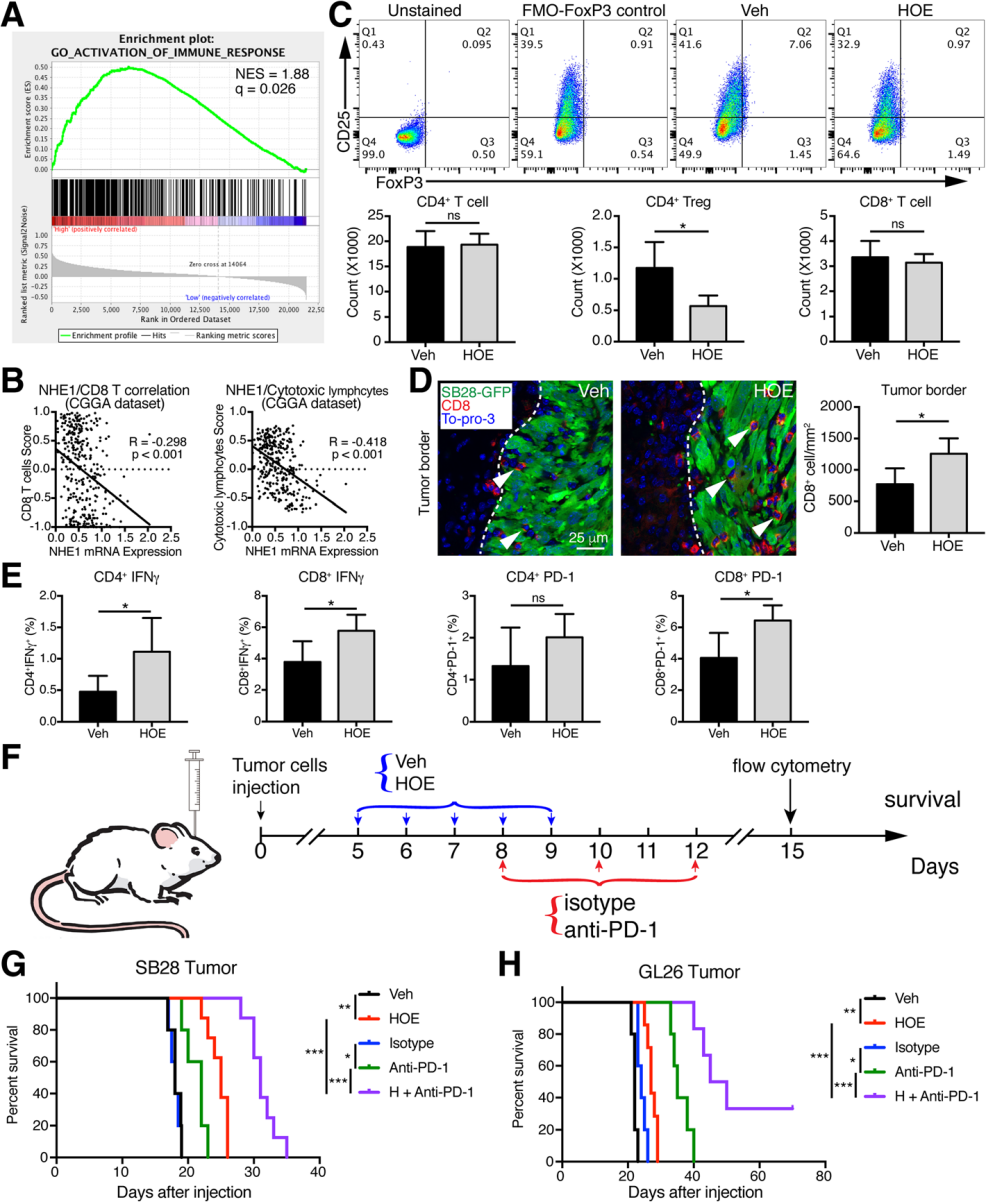
NHE1 blockade enhanced anti-tumor immunity in glioma model. **a** GSEA indicates the diversity of immune phenotype in the case with different NHE1 expression in CGGA dataset. q, adjust *p* value. **b** Regression correlation analysis of NHE1 mRNA expression and CD8 T cells/Cytotoxic lymphocytes metagene score in CGGA dataset. **c** Representative flow-cytometry plots of CD4 T cells stained for CD25 and FoxP3 in unstained (fluorescence minus CD25 and FoxP3), FMO (fluorescence minus FoxP3) control, Veh, and HOE. Data are means ± SD from five independent experiments. ns, no significance; *, *p* < 0.05. **d** Infiltration of CD8 T cell in SB28-GFP tumor borders. Data are means ± SD from four independent experiments. *, *p* < 0.05. **e** Flow cytometric plots of CD4^+^ and CD8^+^ T cells stained for IFNγ and PD-1 are shown in Additional file 1: Figure S9. Data are means ± SD from five independent experiments. ns, no significance; *, *p* < 0.05. **f** Glioma cells were injected into the right striatum of C57BL6/J mice. Starting 5 d.p.i., mice received either vehicle PBS-DMSO (10 ml/kg/day, i.p.) or HOE treatments (0.5 mg/kg/day, i.p.) for 5 consecutive days. Mice received either isotype antibody (10 ml/kg/day, i.p.) or anti-PD-1 (10 mg/kg/day, i.p.) at 8, 10, and 12 d.p.i. **g**, **h** Kaplan-Meier survival curve of SB28-GFP tumor-bearing mice and GL26 tumor-bearing mice, respectively. Each group, *N* = 5–8. *, *p* < 0.05; **, *p* < 0.01; ***, *p* < 0.001

By our GSEA, we unexpectedly found that the cases with various status of *SLC9A1* mRNA expression exhibit different immune phenotypes, and the case with high *SLC9A1* mRNA expression presents enhanced immune phenotypes, compared with low *SLC9A1* mRNA expression ones (Fig. 6a). Meanwhile, we also found that the metagene score of CD8^+^ T cells and cytotoxic lymphocytes are increased in human glioma tissues with decreased *SLC9A1* mRNA expression (Fig. 6b). We then investigated the immune response profiling in the Veh group and HOE642 group of SB28 glioma tumors by flow cytometry. The count of CD4^+^ Treg cells (CD4^+^/CD25^+^/FoxP3^+^) was lower in the tumor with HOE642 treatment, with no changes of CD4^+^ T cells and CD8^+^ T cells (Fig. 6c). More infiltration of CD8^+^ T cells were detected in the tumor border and tumor core of the HOE642-treated tumors (Fig. 6d; Additional file 1: Figure S7). HOE642 treatment reduced the amount of CD11b^+^Gr-1^+^ myeloid cells (Additional file 1: Figure S8A). Moreover, a trend of enhanced *Gzmb* expression in NK1.1^+^ cells was detected in HOE642-treated tumors, although it did not alter the amount of total NK1.1^+^ cells (Additional file 1: Figure S8B and C). In addition, we found an increase in CD4^+^IFNγ^+^ cells and CD8^+^IFNγ^+^ cells in the HOE642-treated SB28 tumors (Fig. 6e; Additional file 1: Figure S9). Consistent with increased IFNγ, we also observed an augment of PD-1 expression in CD4^+^ cells and CD8^+^ cells in the HOE642-treated tumors (Fig. 6e; Additional file 1: Figure S9). These data suggest that inhibition of NHE1 protein reduces immunosuppressive microenvironment but increases the expression of immune checkpoint on the surface of T cells, which prompted us to test combination treatment protocols with immunotherapy of anti-PD-1.

We compared efficacy of HOE642 monotherapy, anti-PD-1 monotherapy or H + anti-PD-1 combination therapy on improving animal overall survival (Fig. 6f). In the case of SB28 tumors, HOE642 monotherapy prolonged the median survival of mice from ~18 days to ~25 days (*p* < 0.05, Fig. 6g). The low dose anti-PD-1 monotherapy did not change median survivals (~22 days vs ~18 days of isotype control). But the combination therapy of HOE642 and anti-PD-1 significantly prolonged the median survival to ~31 days (Fig. 6g). However, in the case of GL26 tumor-bearing mice, the anti-PD-1 monotherapy extended the median survival from ~25 days to ~35 days. The HOE642 plus anti-PD-1 (low dose) therapy increased the median survival to ~48 days (Fig. 6h), 30% of mice in the combination group survived at >90 days of observation time. Taken together, inhibition of NHE1 protein enhanced the anti-tumor immunity and promoted the therapeutic effect of immunotherapy.

Our conclusion is further supported by immune cell profiling analysis of GL26 glioma under the following treatment regimens (Veh, HOE, anti-PD-1, and HOE + anti-PD-1). HOE642 treatment reduced the count of macrophages (CD11b^+^/CD45^high^ cells), while the HOE + anti-PD-1 combination therapy not only increased the TAMs count (Additional file 1: Figure S10A and B), but especially significantly elevated the pro-inflammatory CD16/32^+^ population of TAMs (Additional file 1: Figure S10C) as well as pro-tumoral CD206^+^ population (Additional file 1: Figure S10D). Moreover, the HOE + anti-PD-1 combination therapy significantly reduced the CD4^+^ Treg cell counts (CD4^+^/CD25^+^/FoxP3^+^) in GL26 tumors, with no significant changes of CD4^+^ T cells and CD8^+^ T cells (Additional file 1: Figure S11A and B). In addition, an increase in tumor-infiltration of CD4^+^IFNγ^+^ T cells (*p* < 0.05) and CD8^+^ IFNγ^+^ T cells (*p* = 0.057) was detected in the H + anti-PD-1 combination therapy (Additional file 1: Figure S11C and D). These data clearly indicate that combining anti-PD-1 blockade with NHE1 protein inhibition increases anti-tumor immunity in glioma.

## Discussion

### Elevation of *SLC9A1* in gliomas with poor prognosis outcome

In this study, we further depicted the *SLC9A1* mRNA expression pattern in different grade gliomas from CGGA and TCGA two independent databases. Higher levels of *SLC9A1* mRNA expression were observed in GBM tumors. The classical subtype is defined by the constellation of the most common genomic aberrations, such as chromosome 7 amplifications and 10 deletions, while mesenchymal subtype displayed mesenchymal differentiation [21]. However, both classical and mesenchymal subtype are associated with worse outcome [20, 21]. Here, we detected that *SLC9A1* mRNA expression was higher in the classical and mesenchymal molecular subtypes in CGGA dataset and *SLC9A1* mRNA expression was highest in the mesenchymal molecular subtypes in TCGA dataset. Therefore, higher *SLC9A1* mRNA expression is associated with the malignancy of gliomas. High *SLC9A1* levels of expression showed poor outcomes in each different grade gliomas. Especially, higher *SLC9A1* mRNA expression increased 1.578-fold of hazard ratio (HR), indicating as an independent factor for poor prognosis. This is consistent with the data from the Repository for Molecular Brain Neoplasia Data (REMBRANDT) with shorter overall survival in GBM patients with upregulated *SLC9A1* mRNA expression [15]. Our immunostaining analysis of grade II–III astrocytoma and grade IV gliomas shows that NHE1 protein expression increased with increasing tumor malignancy. Taken together, our analysis (>1000 glioma cases) strongly suggests that NHE1 protein is involved in glioma tumorigenesis and progression. The underlying mechanisms could include promoting tumor proliferation and invasion [37–41]. Our gene ontology analysis indicates that NHE1 protein is involved in the tumor migration and invasion by the regulation of cell adhesion and extracellular matrix organization. NHE1 protein can stimulate tumor cell proliferation via the NHE1-mediated Na^+^ influx for volume increase and H^+^ efflux to maintain alkaline pH_i_ during the cell cycle progression [38]. Pharmacological inhibition of NHE1 has been shown to suppresses the G1/S and G2/M cell cycle phase by the reduction of cell cycle regulator expression, such as cyclin D1 and cyclin B1 [39]. NHE1 protein also stimulates the matrix metalloproteinases family and promote tumor invasion via ERK1/2 and p38 MAPK signaling pathways [42]. In our study, inhibition of NHE1 with HOE642 (0.5 mg/kg per day for continuous 5 days) significantly decreased the tumor volume and tumor invasion in mouse intracranial glioma animal model. HOE642 treatment caused a significant right-shift of survival curve in the glioma-bearing mice.

In both two databases, we found that *SLC9A1* mRNA expression was higher in the IDH-wildtype than in the IDH-mutant gliomas. IDH status plays an important role in the progression of gliomas and the outcome of patients [32, 43]. In a hypoxic environment, cells rely on the reductive carboxylation of glutamine-derived α-ketoglutarate (α-KG) for lipid synthesis and reductive glutamine metabolism is also a characteristic of tumors, including gliomas [44, 45]. The IDH enzymes play a vital role in cellular protection and in response to oxidative and energetic stress via the role of NADPH in the regeneration of the antioxidant glutathione and catalyzing the oxidative carboxylation of isocitrate to α-KG [44, 46]. However, IDH mutant tumor cells downregulate expression of hypoxia-associated genes, such as HIF1α, and increases the risk of reactive oxygen species-mediated DNA damage [44, 47]. NHE1 protein expression is regulated by hypoxia-inducible factor (HIF1α) and facilitates extruding H^+^ to maintain Warburg effects [15, 48]. Therefore, IDH wild-type gliomas may activate NHE1 expression to maintain pH_i_ in response to oxidative stress, promoting tumor cell survival.

### NHE1 is involved in the glioma angiogenesis

Our biological process analysis reveals that high *SLC9A1* mRNA expression was involved in the tumor angiogenesis in both CGGA and TCGA datasets. This result was verified by Gene Set Enrichment analysis. *SLC9A1* mRNA expression was enriched in the tumor area of microvascular proliferation in IVY database. Limited oxygen diffusion and oxygen consumption in the tumor tissue, due to far away from the capillaries, lead to hypoxic tumor microenvironment [48]. The cellular response to the tumor hypoxia is mediated by HIF family, which regulates the gene expression involved in angiogenesis and progression of cancer [49, 50]. Meanwhile, progressive acidification of tumor microenvironment via tumor metabolism and hypoxic tumor microenvironment promote the recruitment of endothelial cells [51]. Suppression of NHE1 by small interfering RNA dramatically decreases the HIF-1α-induced tube formation and migration of endothelial cells in response to hypoxic stress in cultures [52]. We show that the microenvironment cell populations (MCP) score of endothelial cells was positively correlated with the NHE mRNA expression in human glioma tissues. In addition, we detected colocalization and correlation of NHE1 protein and endothelial marker protein CD31 in SB28 glioma tissues. Accumulation of laminin protein and tumor vessel remodeling were inhibited by HOE642 treatment in animal glioma models. Taken together, these findings suggest that NHE1 protein is involved in tumor angiogenesis and suppression of NHE1 expression and function reduces the vascular proliferation and remodeling in the tumor tissues.

### Pharmacological inhibition of NHE1 protein reduced the tumor-associated macrophages and cytokines

Tumor-associated macrophages (TAMs), the major non-neoplastic cells in tumors, are recruited in the glioma microenvironment and promote the tumor proliferation, invasion, angiogenesis and survival by the production of various growth factors and cytokines in response to the factors released by cancer cells [48, 51, 53]. Hypoxia stimulates the accumulation of TAMs, while inhibition of NHE1 expression in glioma cells reduced the stimulation of TAMs in vitro [17, 51]. Here, we found that infiltration of Iba1^+^ cells was inhibited by HOE642 treatment in the intracranial animal glioma model. Meanwhile, low metagene score of infiltrated macrophages was enriched in the cases with low *SLC9A1* mRNA expression. Our flow cytometric profiling study shows that the amount of TAMs was reduced in HOE642 treatment group, with more effects on decreasing resident microglia TAMs than peripheral TAMs. The alternative M2 phenotype of macrophages are suggested to promote the progression of tumor, compared with the pro-inflammatory M1 phenotype of macrophages [53]. It has been reported that lipopolysaccharide (LPS), a ligand of Toll-like receptor (TLR), alters the phenotype of tumor-associated macrophages from M2 to M1 via regulation of NHE1 protein expression in macrophages [54, 55]. In our study, HOE642 treatment inhibited stimulation of M2 phenotypes of TAMs by reducing protumoral CD206^+^ TAMs in both resident microglia TAMs and peripheral TAMs (*p* < 0.05, Fig. 5), and increasing the ratio of M1/M2 TAMs (CD16/32^+^/CD206^+^ or CD86^+^/YM1^+^) in gliomas (*p* = 0.09 and *p* = 0.07, respectively; Additional file 1: Figure S12).

Furthermore, TAM-associated genes were enriched in the tumor area of microvascular proliferation [27]. The main cytokines of TAMs in promoting the proliferation and angiogenesis of tumor [56] were suppressed by HOE642 treatment. Importantly, all cytokines were decreased in resident microglia TAMs in the HOE642-treated tumors. Our findings suggest that inhibition of NHE1 with HOE642 reduces tumor progression and angiogenesis by decreasing the TAMs and associated cytokines, especially resident microglia TAMs. This conclusion is consistent with a report by Brandenburg S et al. [57] that resident microglia TAMs play a critical role in regulation of vascular homeostasis and promotion of angiogenesis.

### Blockade of NHE1 protein enhances the anti-tumor immunity and promotes the immunotherapy of anti-PD-1

Hypoxia creates a functionally immunosuppressive microenvironment by accumulating M2 macrophage and stimulating Treg cell functions [51]. Hypoxic condition promotes expression of immune checkpoint molecules, as such PD-L1 and CTLA-4 [51]. With GSEA, we detected differential immune phenotypes in gliomas with low or high *SLC9A1* mRNA expression. In addition, increased *SLC9A1* mRNA expression is negatively correlated with the infiltration of CD8 T cells (from both human and mouse glioma data). These findings suggest for an involvement of NHE1 protein in the immunosuppressive tumor microenvironment. Treg cells and MDSCs, two vital factors in the immunosuppression of tumor, were reduced in the HOE642-treated tumors, in contrast, cytotoxic T cells were increased in the HOE642-treated tumors, but expression of PD-1 was also increased in T cells. Interestingly, the combination therapy with HOE642 and anti-PD1 significantly extended the survival of glioma-bearing mice, with 30% of GL26 glioma mice survived during the observation period. HOE642 + anti-PD-1 combination therapy reduced the CD4^+^ Treg cell counts while increased tumor infiltration of CD4^+^IFNγ^+^ T cells and CD8^+^ IFNγ^+^ T cells in GL26 tumors. These data further suggest that combining anti-PD-1 blockade with NHE1 protein inhibition increases anti-tumor immunity in glioma.

The poor responses to anti-PD-1 monotherapy is likely due to a low dose (only three treatments, vs routinely eight treatments [58]). We intentionally chose this low dose protocol which allowed us to investigate efficacy of the combinational therapy. However, the survival outcome in the SB28 glioma-bearing mice is worse than GL26 tumors and several factors could play a role, including the weak immunogenicity of SB28 tumors and deficiency of the expressing xenogeneic epitopes, such as CD40 [28]. Additional studies are needed to further optimize treatment duration of HOE642, considering its short half-life (HOE642 ~3.5 h in human serum [59, 60]), and doses of anti-PD1 antibody to reduce immunosuppressive microenvironment of tumor and enhance the anti-PD1 tumor immunity.

## Conclusions

In summary, this is the first study to delineate the molecular and clinical characteristics of *SLC9A1* mRNA expression in gliomas. In this study, we found that high *SLC9A1* mRNA was associated with malignancy of gliomas and predicted a poor prognosis of glioma patients. Moreover, genes positive with *SLC9A1* mRNA expression were mainly involved in tumor invasion and angiogenesis. Pharmacological blockage of NHE1 protein reduced the tumor volume, invasion and angiogenesis in intracranial syngeneic mouse glioma models. Interestingly, inhibition of NHE1 protein with HOE642 decreased the accumulation of tumor-associated macrophages and cytokine secretion, but enhanced the anti-tumor immunity and immunotherapy of anti-PD-1 in mouse glioma models. Therefore, NHE1 expression may serve as a biomarker for the tumorigenesis and progression of gliomas. Blockade of NHE1 protein presents a novel strategy for combinational glioma therapy.

## Additional files

Additional file 1: **Supplemental Methods.** Immunostaining image analysis, Cell counting, Fluorescence intensity quantification. **Figure S1.** Representative images of secondary antibody only staining. **Figure S2.** Differential NHE1 protein expression in human gliomas. **Figure S3.** SLC9A1 mRNA expression is related with angiogenesis in gene ontology analysis in TCGA dataset. **Figure S4.** Inhibition of NHE1 decreases the tumor vessel density in SB28 gliomas. **Figure S5.** HOE642 treatment decreases the resident-microglia TAMs and bone-marrow-derived TAMs in SB28 gliomas. **Figure S6.** Inhibition of NHE1 decreases the cytokines of TAMs in SB28 gliomas. **Figure S7.** Blockade of NHE1 increases the CD8 T cell infiltration. **Figure S8.** HOE642 treatment reduced the accumulation of MDSC in SB28 gliomas. **Figure S9.** Inhibition of NHE1 increases T cell anti-tumor immunity in SB28 glioma model. **Figure S10.** HOE642 plus anti-PD-1 combination therapy increases the tumor-associated macrophages infiltration in GL26 gliomas. **Figure S11.** HOE plus anti-PD-1 combination therapy stimulates T cell immunity in GL26 tumor. **Figure S12.** The effect of HOE642 treatment on the ratio of M1/M2 tumor-associated macrophages. (DOCX 3855 kb)

Additional file 2 **Table S1.** The information of human glioma tissue (DOCX 23 kb)

## Abbreviations

CGGA: Chinese Glioma Genome Atlas
CL: Classical
GBM: Glioblastoma
GSEA: Gene set enrichmental analysis
GSVA: Gene set variation analysis
IDH Mut: IDH-mutant
IDH WT: IDH-wildtype
IDH: Isocitrate dehydrogenase
ME: Mesenchymal
NE: Neural
NHE1: Sodium/hydrogen exchanger 1
pH_i_: Intracellular pH
PN: Proneural
*SLC9A1*: SoLute Carrier family 9A1
TAM: Tumor associated macrophage
TCGA: The Cancer Genome Atlas
