# Supplementary material for: Elevated Na/H exchanger 1 (SLC9A1) emerges as a marker for tumorigenesis and prognosis in gliomas
Source: J Exp Clin Cancer Res. 2018 Oct 17;37:255. doi: 10.1186/s13046-018-0923-z (PMC6192309; doi:10.1186/s13046-018-0923-z)
Supplement: Supplementary file 1 — Supplemental Methods. Immunostaining image analysis, Cell counting, Fluorescence intensity quantification. Figure S1. Representative images of secondary antibody only staining. Figure S2. Differential NHE1 protein expression in human gliomas. Figure S3. SLC9A1 mRNA expression is related with angiogenesis in gene ontology analysis in TCGA dataset. Figure S4. Inhibition of NHE1 decreases the tumor vessel density in SB28 gliomas. Figure S5. HOE642 treatment decreases the resident-microglia TAMs and bone-marrow-derived TAMs in SB28 gliomas. Figure S6. Inhibition of NHE1 decreases the cytokines of TAMs in SB28 gliomas. Figure S7. Blockade of NHE1 increases the CD8 T cell infiltration. Figure S8. HOE642 treatment reduced the accumulation of MDSC in SB28 gliomas. Figure S9. Inhibition of NHE1 increases T cell anti-tumor immunity in SB28 glioma model. Figure S10. HOE642 plus anti-PD-1 combination therapy increases the tumor-associated macrophages infiltration in GL26 gliomas. Figure S11. HOE plus anti-PD-1 combination therapy stimulates T cell immunity in GL26 tumor. Figure S12. The effect of HOE642 treatment on the ratio of M1/M2 tumor-associated macrophages. (DOCX 3855 kb) [file 13046_2018_923_MOESM1_ESM.docx]

**Additional file 1**

**Supplemental Methods**

**Immunostaining image analysis**

Confocal images of immune stained brain sections (25 μm) were acquired with a Leica DMIRE2 inverted confocal laser scanning microscope, using a 40x objective, identical pinhole, intensity, and exposure parameters were applied for all the images to be compared. Images were computer processed in Image J (National Institutes of Health), Adobe photoshop CC and Adobe illustrator softwares. Background intensity in each original image was subtracted using Image J software as described previously [1].

**Cell counting**: In a blinded manner, positively stained cells (iba1, CD8) were counted in the tumor or in the tumor border of coronal brain sections (n=4) using ImageJ Cell Counter plugin. The positive signal localized to the cytoplasmic puncta was considered for counting the Iba1 and CD8 positive immunosignals. For negative controls, brain sections were stained with secondary antibody only. In each section, 4-6 randomly selected fields were analyzed and averaged per brain section. The number of cells that were positively stained with anti-Iba1 or anti-CD8 in each area was normalized and expressed as immunolabeled cells relative to the tumor area assessed by GFP in each area.

**Fluorescence intensity quantification:** To quantify NHE1 or CD31 expression, representative images from 4-6 regions per brain section (n=4) were taken under identical camera and microscope settings. For every image, the mean intensity values were measured using Image J software.

**Figure S1**


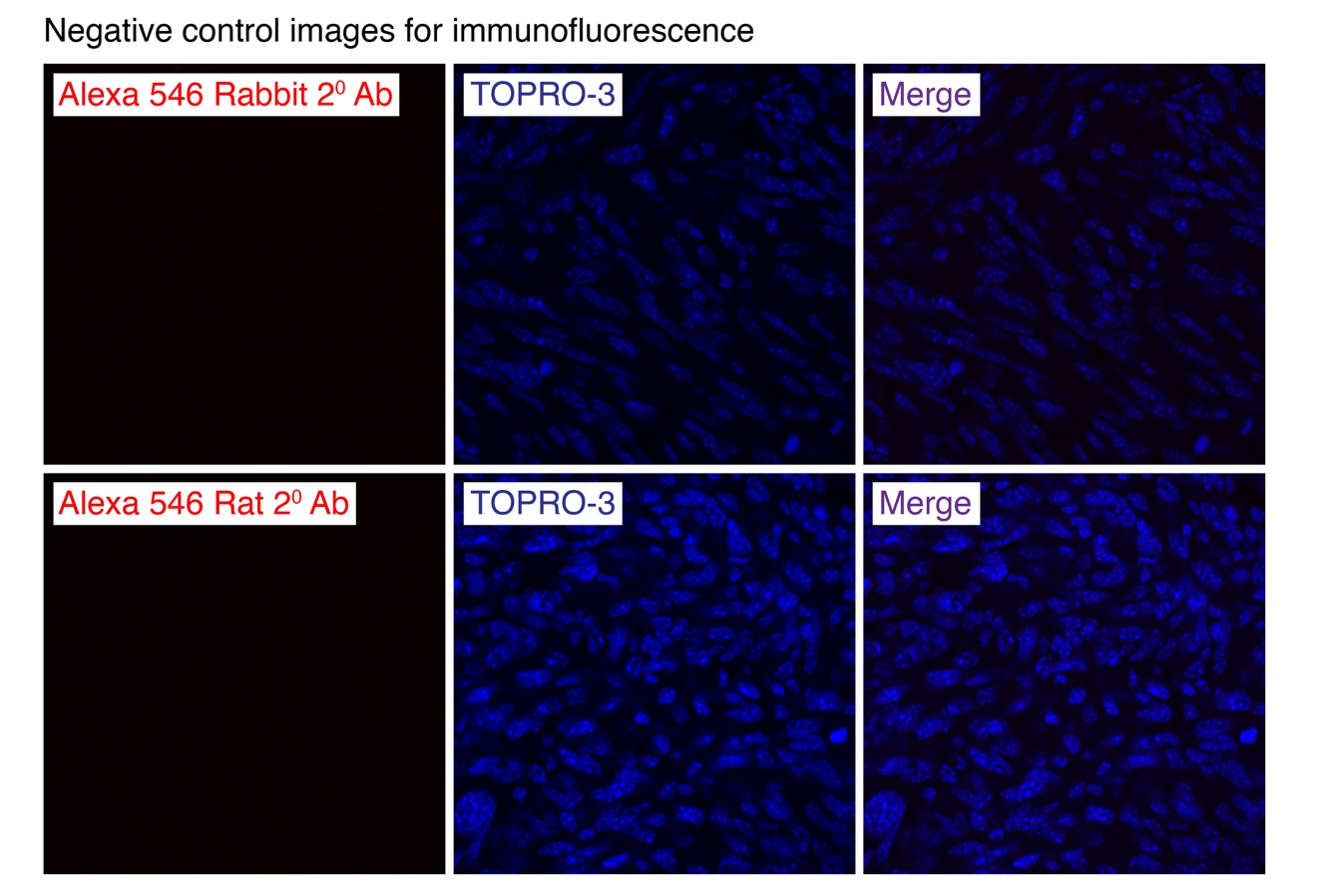


**Fig S1. Representative images of secondary antibody only staining (Alexa 546 rabbit secondary or Alexa 546 rat secondary antibody) in the tumor core.**

**Figure S2**


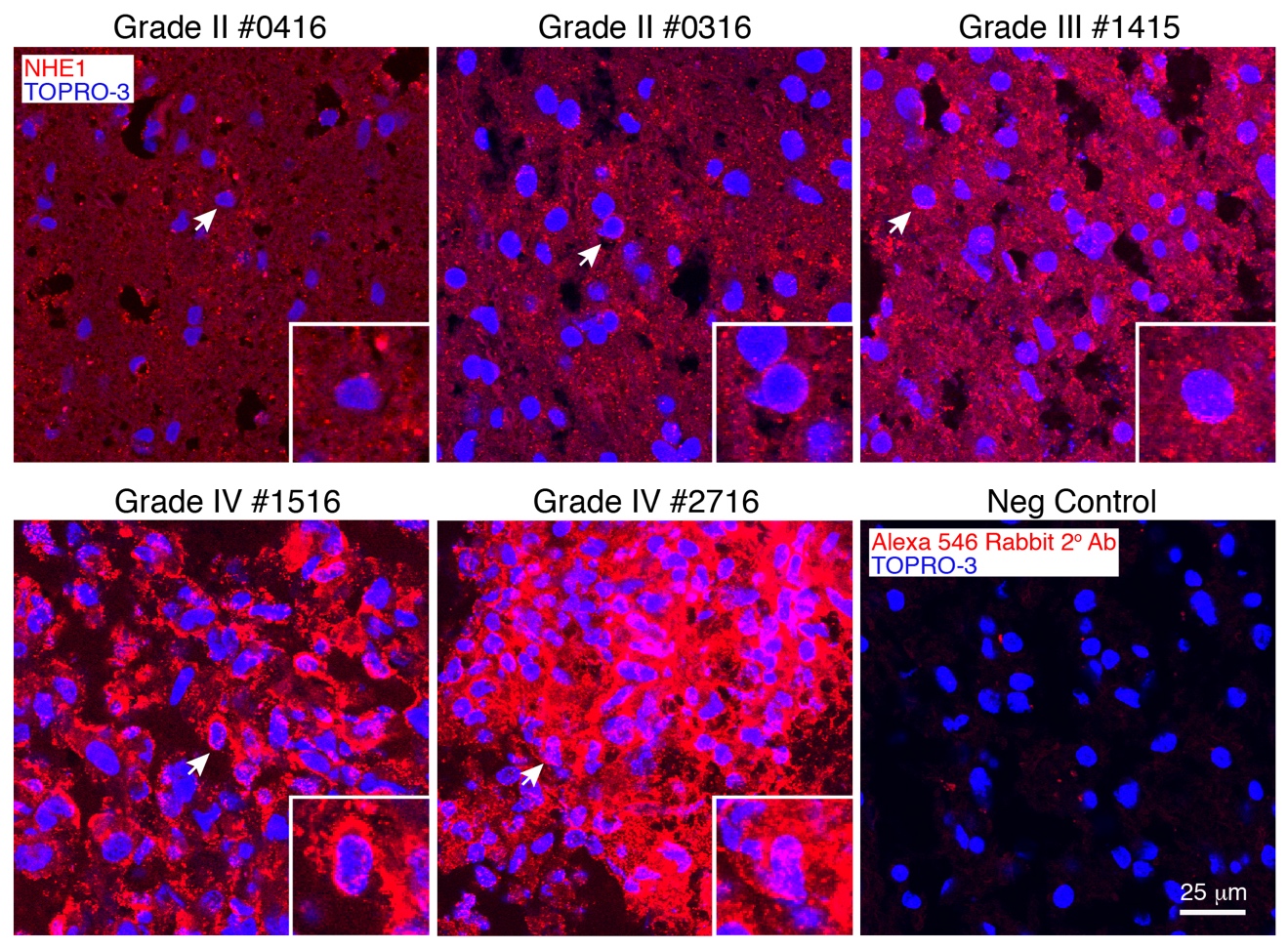


**Fig S2. Differential NHE1 protein expression in human gliomas.**

Representative immunofluorescence staining images of NHE1 protein show that NHE1 immunoreactive fluorescence signal intensity (**arrows**) was increased with increasing malignancy of tumors among Grade II-IV glioma tissues (paraformaldehyde-fixed 25 μm sections). Negative control images were obtained in tumor tissue sections when only secondary antibody was present. The WHO grade II-IV glioma information of patients was shown in **Supplementary Table 1**.

**Figure S3**


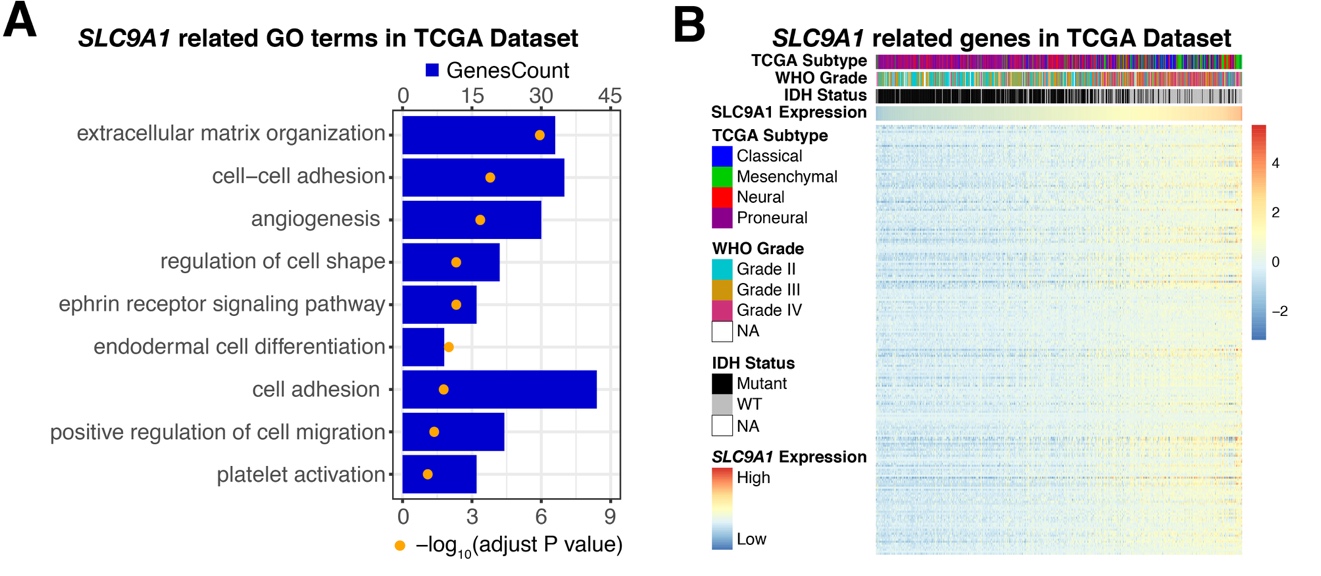


**Fig S3. *SLC9A1* mRNA expression is related with angiogenesis in gene ontology analysis in TCGA dataset.**

**A.** Gene ontology (GO) analysis of positively related biological process in TCGA dataset also shows that NHE1-associated genes are enriched in extracellular matrix organization, angiogenesis and cell adhesion. **B.** The heat maps display *SLC9A1*-related genes in TCGA cohorts.

**Figure S4**


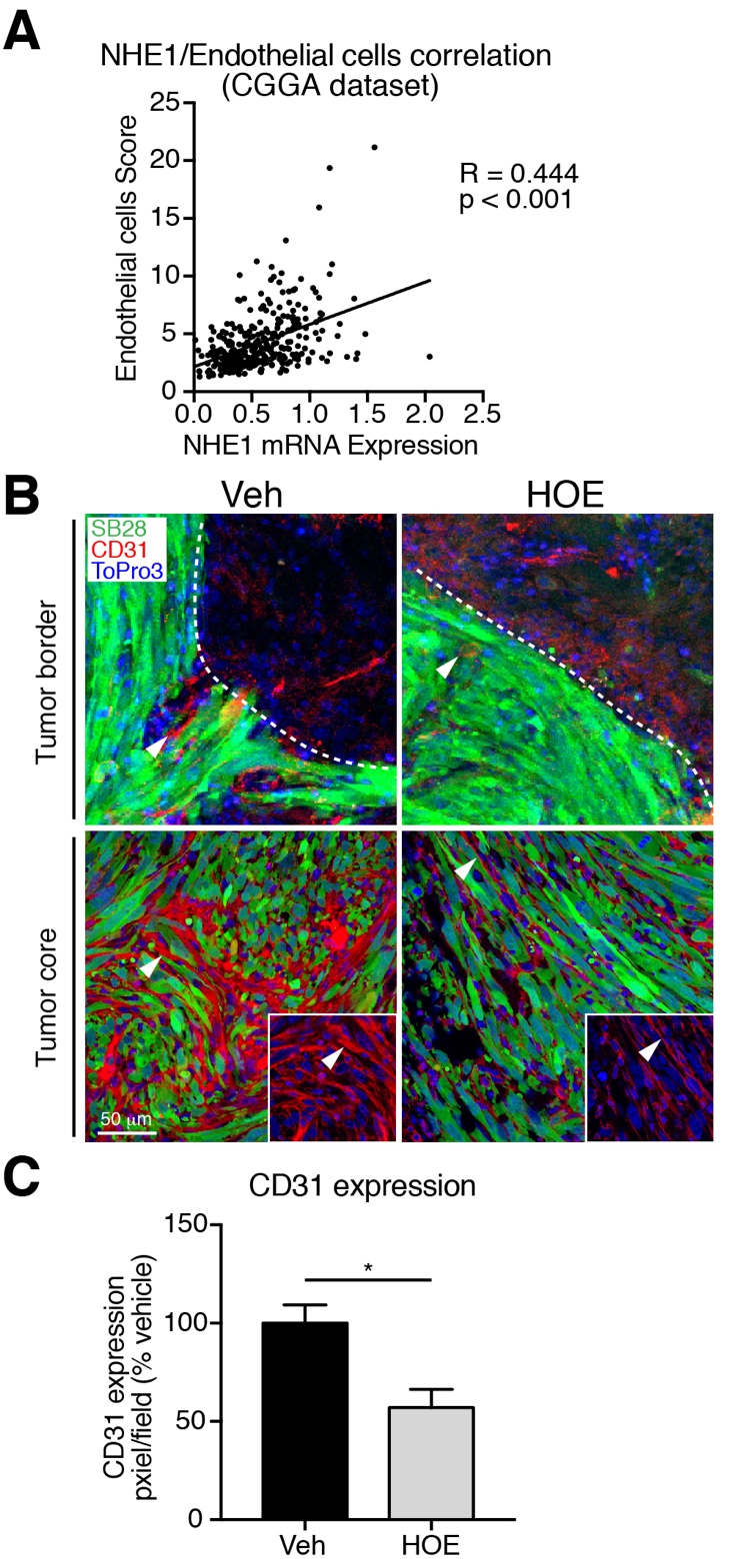


**Fig S4. Inhibition of NHE1 decreases the tumor vessel density in SB28 gliomas.**

**A.** Regression correlation analysis of NHE1 mRNA expression and endothelial cells MCP-Score in CGGA dataset. **B.** Representative immunostaining of fixed brain sections (25 μm) for blood vessels in the tumor borders and cores of SB28-GFP gliomas. **C.** Data summary by vessel area/total area and CD31 expression. Data are means ± SEM from four independent experiments. *, p<0.05.

**Figure S5**


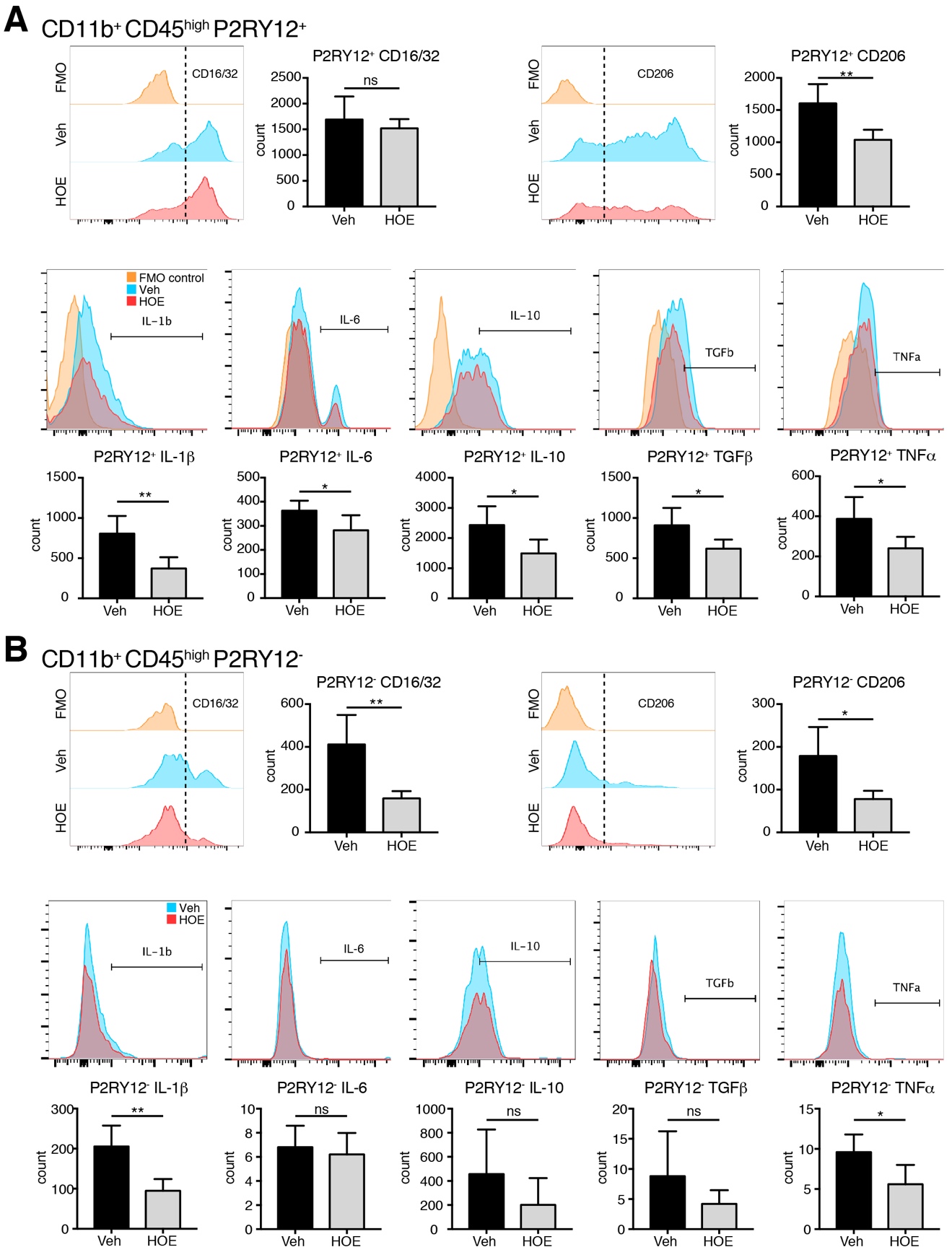


**Fig S5. HOE642 treatment decreases the resident-microglia TAMs and bone-marrow-derived TAMs in SB28 gliomas.**

**A.** Representative flow-cytometry plot of resident-microglia TAMs (CD11b^+^/CD45^high^/P2RY12^+^) stained for CD16/32, CD206, and cytokines in SB28 tumors. **B.** Representative flow-cytometry plot of bone marrow-derived TAMs (CD11b^+^/CD45^high^/P2RY12^+^) stained for CD16/32, CD206, and cytokines in SB28 tumors.

**Figure S6**


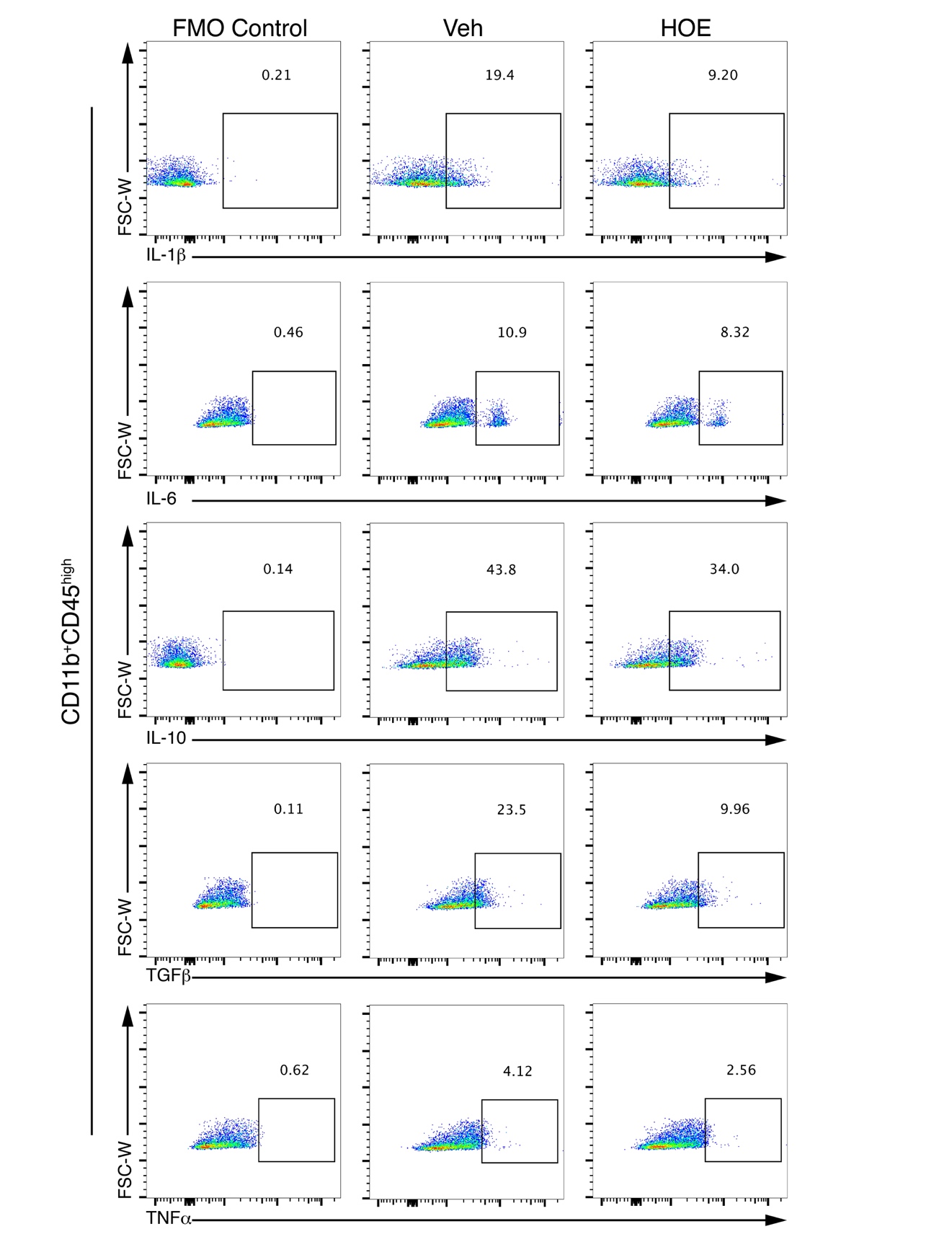


**Fig S6. Inhibition of NHE1 decreases the cytokines of TAMs in SB28 gliomas.**

Representative flow-cytometry plot of TAMs stained for cytokines (IL-1β, IL-6, IL-10, TGFβ, and TNFα) in fluorescence minus one (FMO) control-, Veh-, and HOE642-treated SB28 tumors.

**Figure S7**


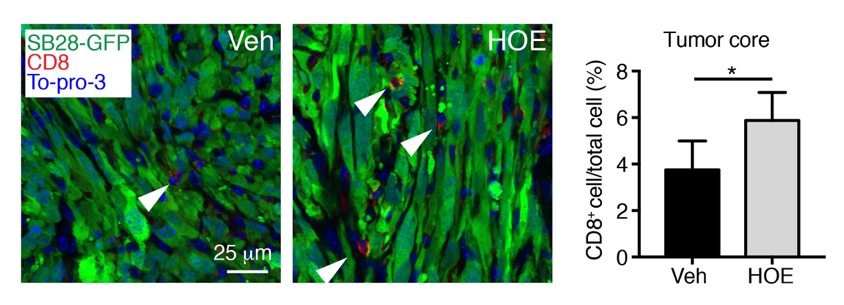


**Fig S7. Blockade of NHE1 increases the CD8 T cell infiltration.**

Infiltration of CD8^+^ cells in SB28-GFP tumor cores. Data are means ± SD from five independent experiments. *, p<0.05

**Figure S8**


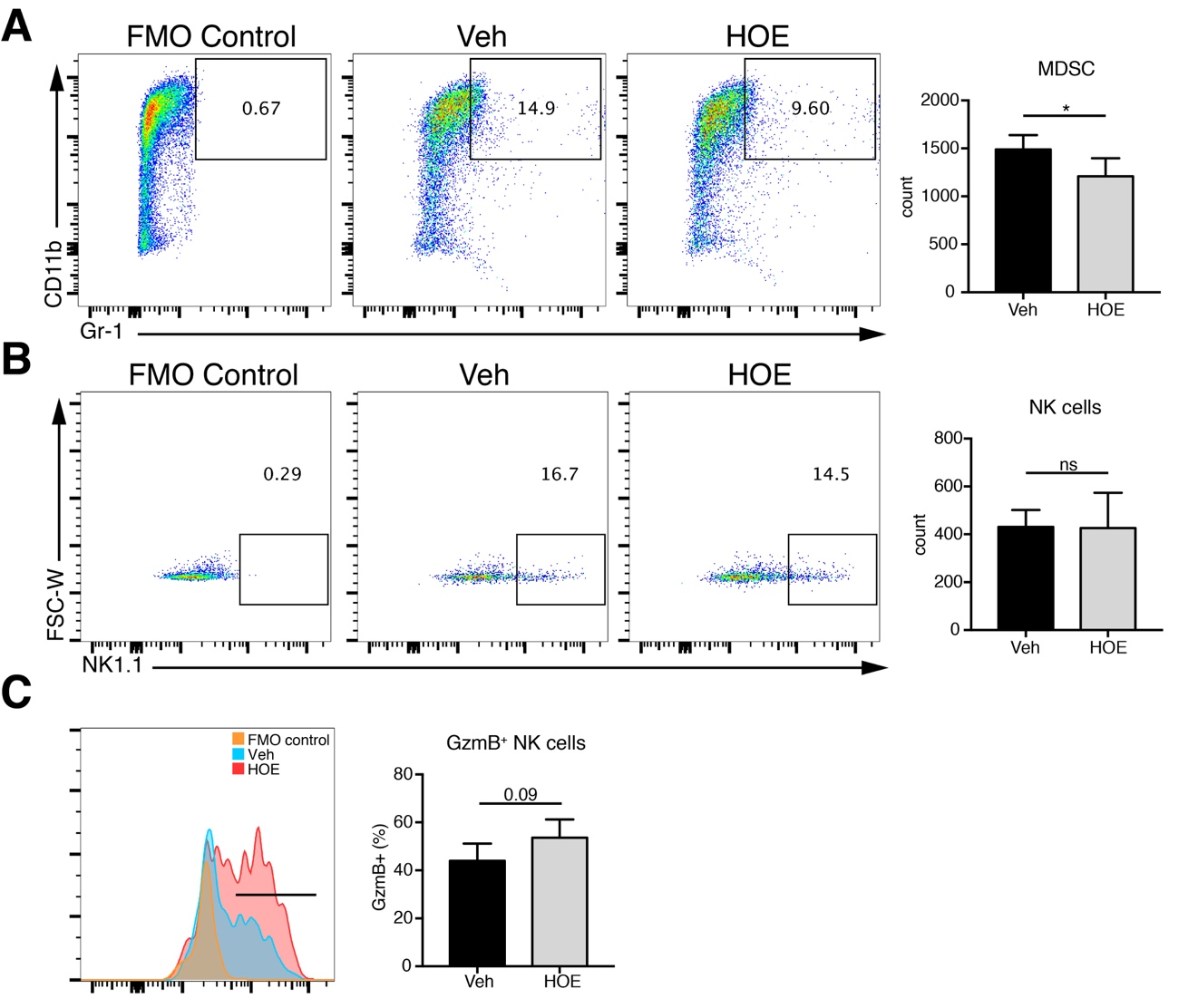


**Fig S8. HOE642 treatment reduced the accumulation of MDSC in SB28 gliomas.**

**A.** Flow cytometric profiles of CD11b^+^/Gr-1^+^ myeloid cells (gated on CD45+) in fluorescence minus one (FMO) control-, Veh-, and HOE642-treated SB28 tumors. N=4-5 per group. *, p<0.05. **B.** Flow cytometric profiles of CD11b^low^/NK1.1^+^ cells (gated on CD45+). N=4-5 per group. ns, no significance. **C.** Flow cytometric profiles of CD11b^low^/NK1.1^+^ cells stained for GzmB. N=4-5 per group.

**Figure S9**


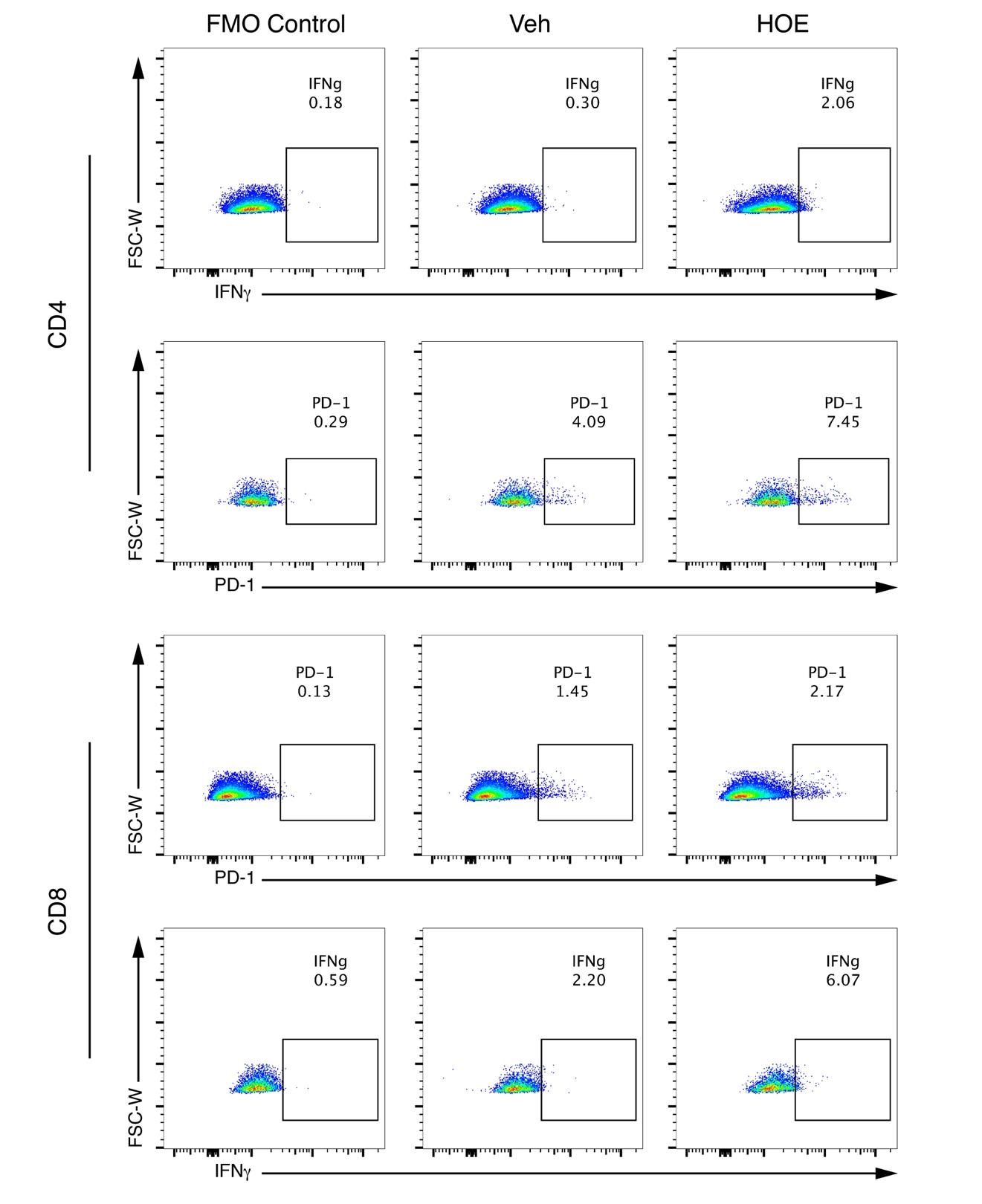


**Fig S9. Inhibition of NHE1 increases T cell anti-tumor immunity in SB28 glioma model.**

Flow cytometric profiles of CD4^+^ and CD8^+^ T cells stained for IFNγ and PD-1 in fluorescence minus one (FMO) control-, Veh-, and HOE642-treated SB28 tumors.

**Figure S10**


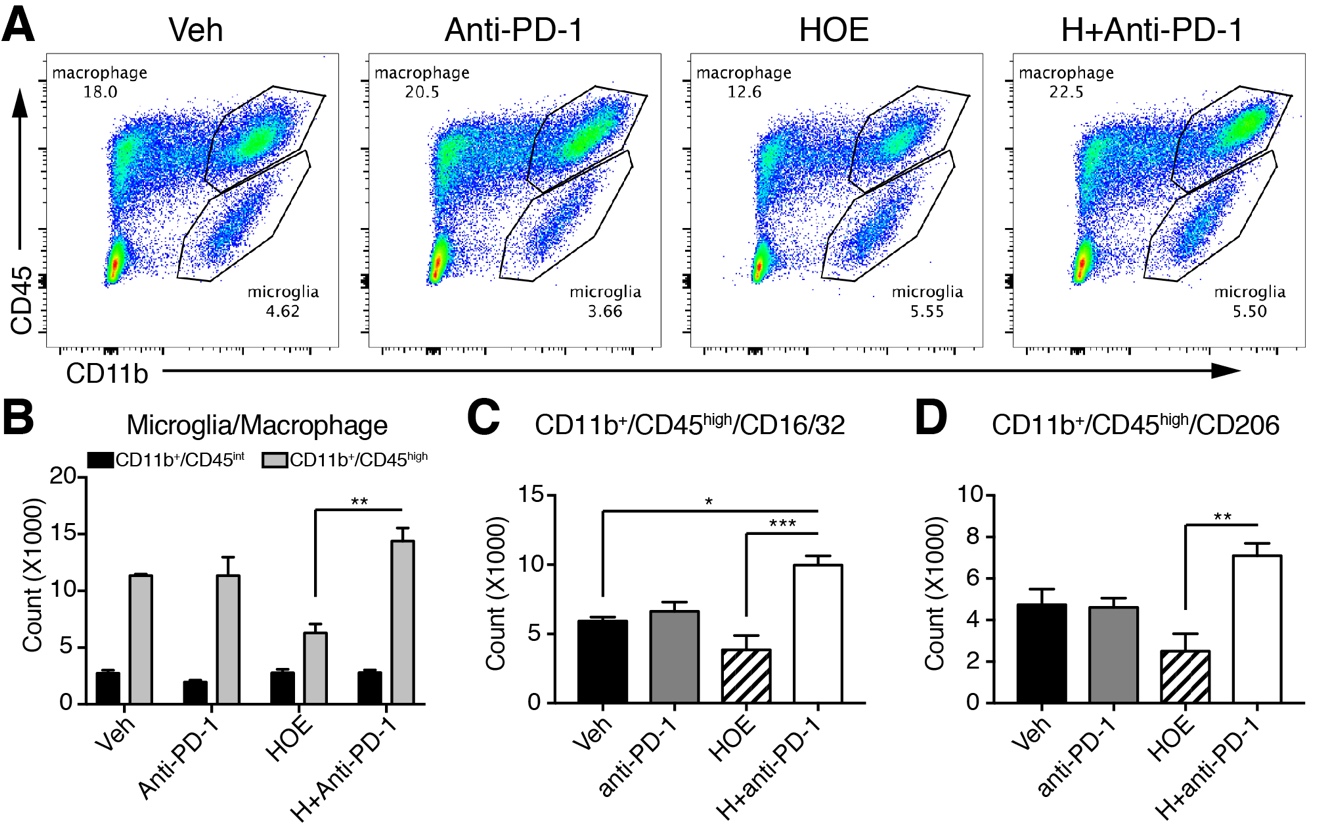


**Fig S10. HOE642 plus anti-PD-1 combination therapy increases the tumor-associated macrophages infiltration in GL26 gliomas.**

**A.** Representative flow cytometric profile showing gating strategy of microglia (CD11b^+^/CD45^int^) and macrophages (CD11b^+^/CD45^high^) in GL26 glioma in the same protocol as described in **Figure 6F**. **B.** Data summary of TAMs. Data are means ± SD from four independent experiments. **, p<0.01. **C, D.** Inflammatory profile of TAMs stained for CD16/32 and CD206. Data are means ± SD from four independent experiments. *, p<0.05; **, p<0.01.

**Figure S11**


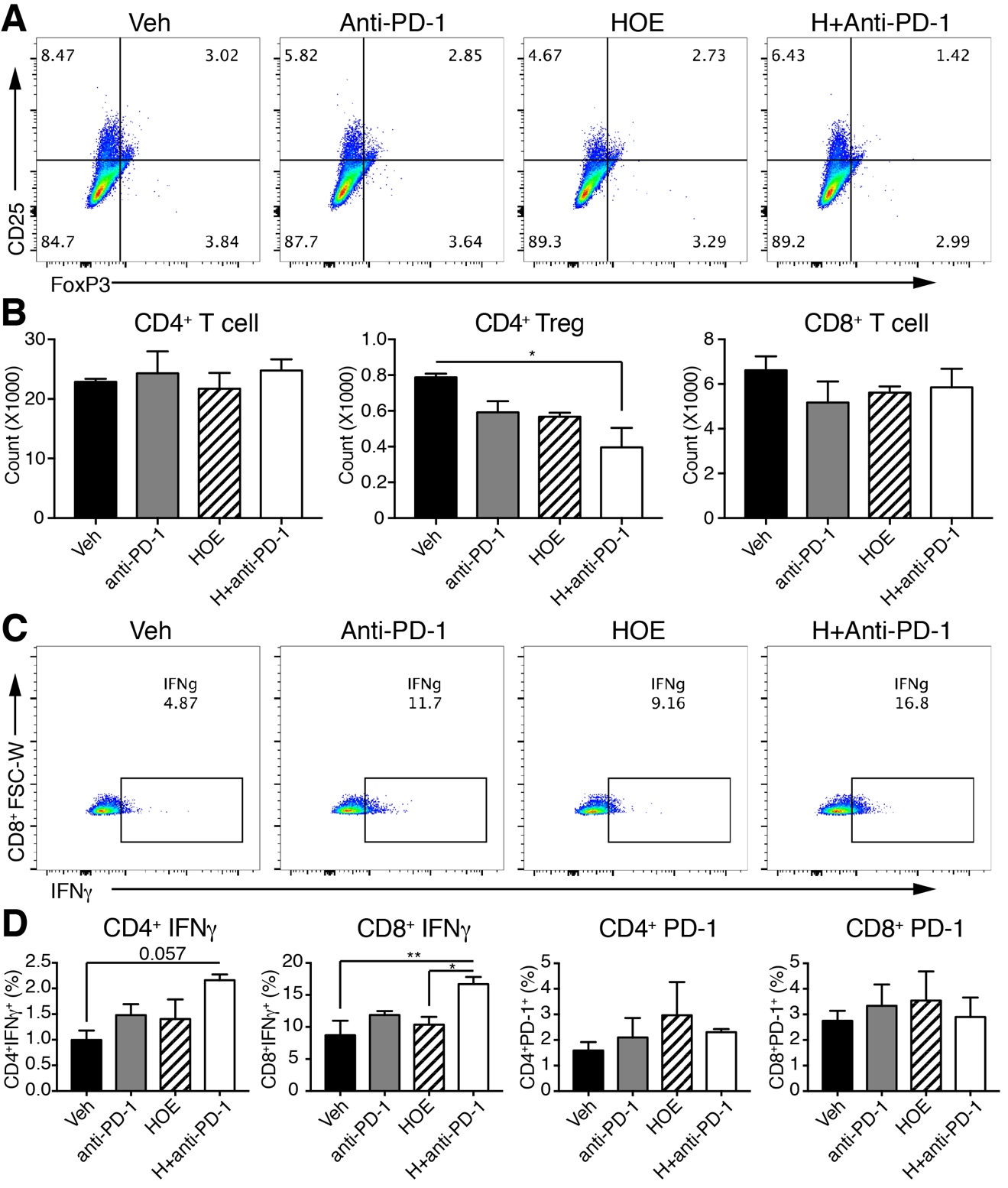


**Fig S11. HOE plus anti-PD-1 combination therapy stimulates T cell immunity in GL26 tumor.**

**A.** Representative flow-cytometry plots of CD4 T cells stained for CD25 and FoxP3 in the Veh-, anti-PD-1-, HOE-, and HOE + anti-PD-1 treatment regimens. **B.** Inflammatory profile of CD4^+^ T cell, CD4^+^ Treg, and CD8^+^ T cell. Data are means ± SD from four independent experiments. *, p<0.05. **C.** Representative flow-cytometry plots of CD8^+^ T cells stained for IFNγ in the Veh-, anti-PD-1-, HOE-, and HOE + anti-PD-1 treatment regimens. **D.** Inflammatory profiles of CD4^+^ T cell and CD8^+^ T cells stained for IFNγ and PD-1. *, p<0.05; **, p<0.01.

**Figure S12**


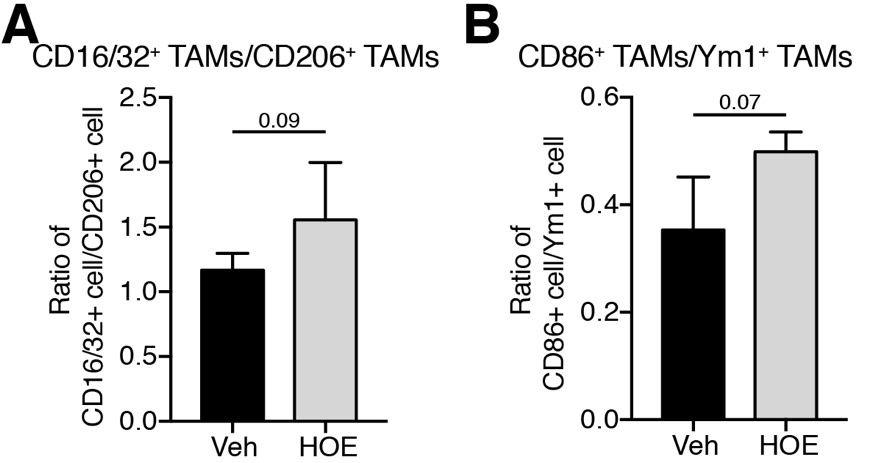


**Fig S12. The effect of HOE642 treatment on the ratio of M1/M2 tumor-associated macrophages.**

**A.** The ratio of CD16/32^+^ TAMs/CD206^+^ TAMs in SB28 glioma. **B.** The ratio of CD86^+^ TAMs/CDYm1^+^ TAMs in SB28 glioma. Data are mean ± SEM from three independent experiments.

**References**

1. Begum G, Yuan H, Kahle KT, Li L, Wang S, Shi Y, Shmukler BE, Yang SS, Lin SH, Alper SL, Sun D: **Inhibition of WNK3 Kinase Signaling Reduces Brain Damage and Accelerates Neurological Recovery After Stroke.** *Stroke* 2015, **46:**1956-1965.
