# Supplementary material for: Elevated Na/H exchanger 1 (SLC9A1) emerges as a marker for tumorigenesis and prognosis in gliomas
Source: J Exp Clin Cancer Res. 2018 Oct 17;37:255. doi: 10.1186/s13046-018-0923-z (PMC6192309; doi:10.1186/s13046-018-0923-z)
Supplement: Supplementary file 2 — Table S1. The information of human glioma tissue (DOCX 23 kb) [file 13046_2018_923_MOESM2_ESM.docx]

| **Additional file 2: Table S1. The information of human glioma tissue** | | | |
| --- | --- | --- | --- |
| Database  number | WHO Grade | Histological diagnosis | IDH status |
| #0416 | Grade II | Astrocytoma | Mutant |
| #0316 | Grade II | Astrocytoma | Mutant |
| #1415 | Grade III | Astrocytoma | Mutant |
| #1516 | Grade IV | GBM | Wild-type |
| #2716 | Grade IV | GBM | Wild-type |
